# Evolution characteristics and causes of iodine and fluoride in groundwater of Hengshui city in North China

**DOI:** 10.1038/s41598-024-83601-2

**Published:** 2024-12-30

**Authors:** Yapeng Tuo, Baizhong Yan, Junbai Gai, Yanbo Yu, Xinkai Zhan, Yuanjing Zhang, Shuwei Qiu

**Affiliations:** 1 Hebei Province Collaborative Innovation Center for Sustainable Utilization of Water Resources and Optimization of Industrial Structure, Hebei GEO University, Shijiazhuang, 050031 China; 2 Hebei Province Key Laboratory of Sustained Utilization & Development of Water Resources, Hebei GEO University, Shijiazhuang, 050031 China; 3 Hebei Center for Ecological and Environmental Geology Research, Hebei GEO University, Shijiazhuang, 050031 China; 4 Institute of Hydrogeology and Environmental Geology, Chinese Academy of Geological Sciences, Shijiazhuang, 050061 Hebei China

**Keywords:** Groundwater funnel area, Iodine, Fluoride, Evolution characteristics, Groundwater exploitation reduction, Ecology, Environmental sciences, Environmental social sciences, Hydrology

## Abstract

Iodine and fluoride are essential trace elements for human health, with both deficiency and excess intake impacting well-being. This study investigates the groundwater funnel area in eastern Hengshui City, utilizing groundwater level and hydrochemical data from 2014 to 2022. Hydrogeochemical methods were employed to comprehensively analyze the evolution characteristics and causes of iodine and fluoride concentrations in the funnel area. The results show: (1) After the implementation of groundwater exploitation reduction (GER) (post-2014), the mean concentration of I⁻ in the study area’s shallow groundwater (SG) decreased from 0.17 mg/L to 0.16 mg/L. Conversely, the mean concentration of F⁻ increased from 1.00 mg/L to 1.12 mg/L. In the deep groundwater (DG), the mean concentration of I⁻ rose from 0.17 mg/L to 0.19 mg/L, and the mean concentration of F⁻ increased from 1.99 mg/L to 2.90 mg/L. (2) In 2014, the concentrations of I⁻ and F⁻ in SG increased progressively from the recharge area to the discharge area along the groundwater flow. By 2018 and 2022, the concentrations of I⁻ and F⁻ in the recharge area had become higher than those in the discharge area. Between 2014 and 2022, the concentrations of I⁻ and F⁻ in DG progressively increased from the recharge area to the discharge area along the groundwater flow. (3) Before and after GER, the primary sources of I⁻ and F⁻ in both SG and DG remained consistent. Nevertheless, prolonged GER and recharge have altered the groundwater hydraulic conditions, pH, redox environment, HCO₃⁻ concentration, Ca^2+^ concentration, and cation exchange processes. These changes have led to the evolution of I⁻ and F⁻ concentrations.

**Supplementary Information:**

The online version contains supplementary material available at 10.1038/s41598-024-83601-2.

## Introduction

Iodine and fluoride are essential trace elements for human health, but both excessive and insufficient intake can negatively affect the body^1,2^. Numerous studies have shown that excessive intake of iodine and fluoride from drinking water is a major cause of endemic goiter and dental fluorosis^3–5^. Given that groundwater is a globally important drinking water resource^6^, understanding the evolution and causes of iodine and fluoride in groundwater is crucial.

In recent years, large-scale and long-term over-exploitation of groundwater has led to the formation of numerous groundwater funnel areas, significantly limiting the development and use of groundwater resources^7,8^. Many countries have implemented comprehensive measures to manage groundwater over-exploitation. In 2014, the California government promulgated the “Sustainable Groundwater Management Act”^9^. In order to effectively deal with the problem of groundwater over-exploitation and long-term decline of water level, the Government of India has launched a nationwide project “Aquifer Mapping and Management”^10^. In 2000, the European Union issued the “Water Framework Directive” for the protection of groundwater resources^11^. The Chinese government has been scrambling since 2014 to develop a series of measures to address the problem of over-exploitation of groundwater in North China. In 2019, the Ministry of Water Resources of the People’s Republic of China formulated the Action Program for “Action Plan for Comprehensive Management of Groundwater Over-exploitation in North China” proposing to systematically promote the management of groundwater over-exploitation in North China by adopting the comprehensive management measures of “one reduction, one increase”^12^. The decline in groundwater levels has been effectively controlled, with some areas experiencing partial recovery in water levels^13^. As groundwater hydrodynamic conditions change, the hydrochemical characteristics of the groundwater will also be altered^14^. The migration and enrichment of I⁻ and F⁻ are strongly influenced by the groundwater’s hydrochemical environment^15^. Kang et al.^16^ indicated that the primary sources of chemical components in high-iodine groundwater is the weathering of silicates and evaporites. Zhang et al.^17^ and Qian et al.^18^ investigated the migration and enrichment of iodine in groundwater by analyzing its hydrochemical characteristics, occurrence environment, and hydrogeochemical processes. They identified that a mildly alkaline and weakly reducing environment, groundwater flow intensity, and the competitive adsorption between HCO₃⁻ and I⁻ are crucial factors influencing iodine migration and enrichment. Su et al.^19^ analyzed the sources and causes of fluoride in high-fluoride groundwater. Their research revealed that the weathering and dissolution of fluorite and other fluoride-bearing minerals are the primary sources of F⁻ in groundwater. Cao et al.^20^ and Wang et al.^21^ analyzed the distribution and causes of fluoride enrichment in groundwater. They identified that factors like groundwater hydraulic conditions, TDS, pH, redox environment, HCO₃⁻ concentration, and Ca²⁺ concentration promote the migration and enrichment of F⁻ in groundwater by influencing the dissolution of fluoride-bearing minerals, evaporation concentration, and ion exchange processes. Current research on high-iodine and high-fluoride groundwater primarily focuses on distribution, migration, and co-enrichment mechanisms. However, there is a lack of studies on the evolution of iodine and fluoride in groundwater within funnel areas, especially under the environmental changes induced by comprehensive management measures aimed at groundwater over-exploitation.

Hengshui City, situated in the North China Plain, relies heavily on groundwater as its primary water source and has significant extraction levels. It is a typical representative of a groundwater funnel area^22^. Currently, in regions with naturally high-iodine and high-fluoride groundwater^23,24^, the patterns of iodine and fluoride migration and enrichment remain unclear, under environmental changes caused by groundwater level recovery following extraction reduction. This study aims to analyze the hydrochemical data of groundwater samples collected from the eastern funnel area of Hengshui City from 2014 to 2022. Incorporating the geological background, hydrogeological conditions, and the influence of human activities, it investigates the evolution characteristics and causes of iodine and fluoride in groundwater. This can provide reference for the study of local drinking water safety and the solution of endemic diseases.

## Materials and methods

### Study area

**Fig. 1:**
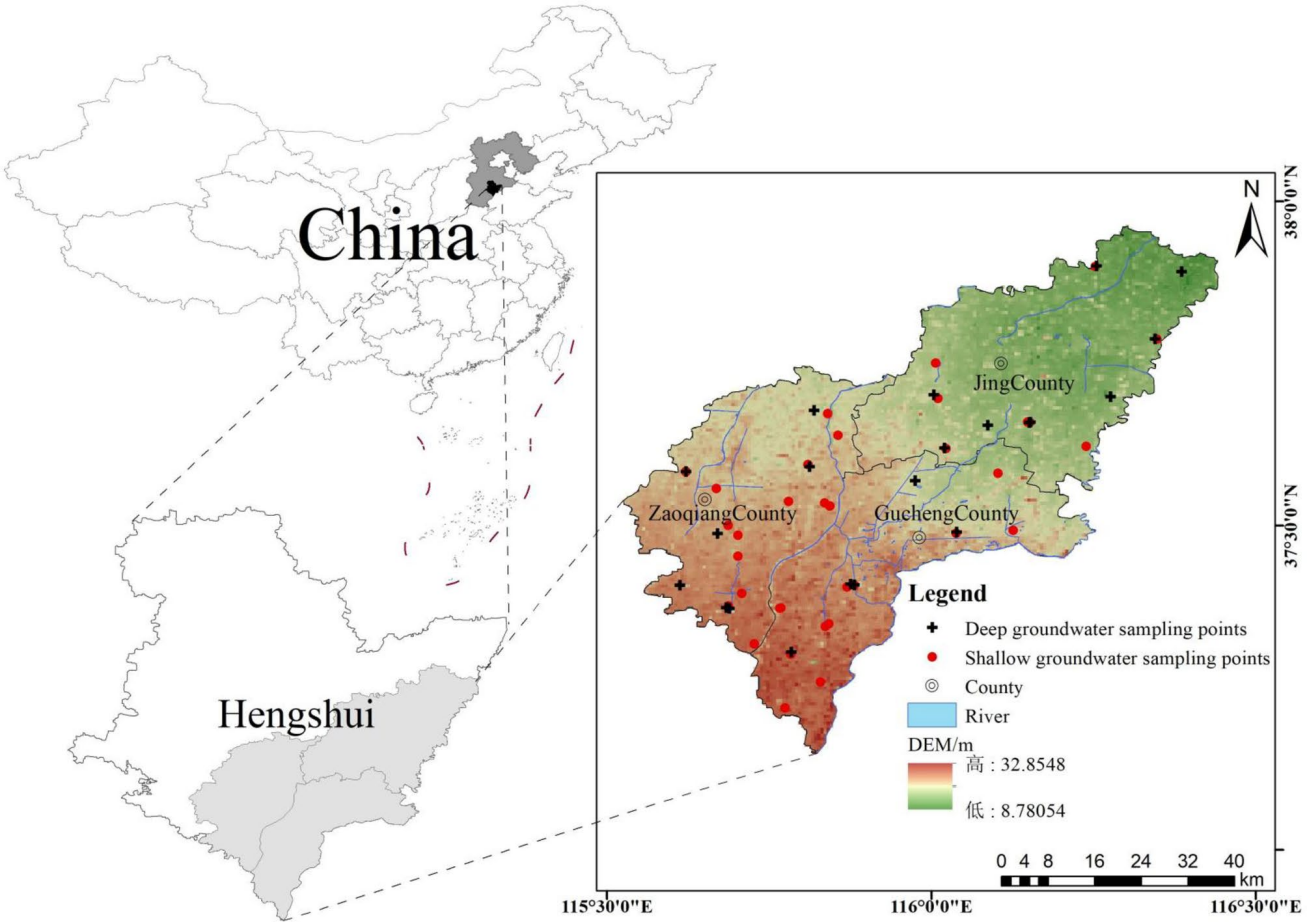
Distribution of groundwater quality sampling points in the study area. The map was created via ArcGIS 10.8.1 (https://www.esri.com/en-us/arcgis/products/arcgis-desktop/resources) based on the standard map No. GS(2019)1822 from the Standard Map Service of the Ministry of Natural Resources ( http://211.159.153.75/ ). No modiffcations were made to the base map.

**Fig. 2:**
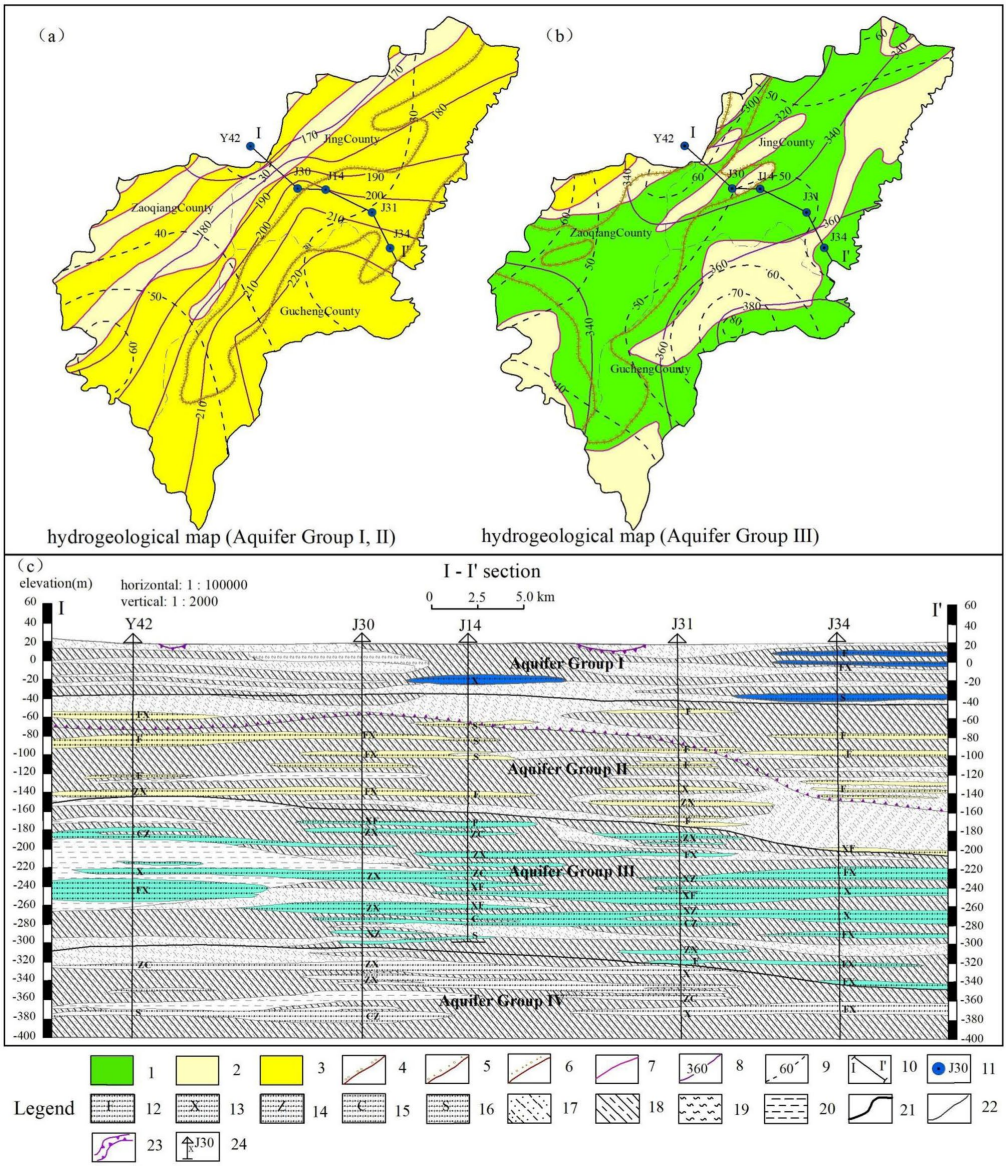
Hydrogeological map and section map. [1: Specific yield of single well 300–500 m³/(d·m); 2: Specific yield of single well 100–300 m³/(d·m); 3: Specific yield of single well < 100 m³/(d·m); 4: The distribution area of aquifers mainly composed of medium sand (More than 50% of the total thickness of the aquifer); 5: The distribution area of aquifers mainly composed of fine sand (More than 50% of the total thickness of the aquifer); 6: The distribution area of aquifers mainly composed of silt (More than 50% of the total thickness of the aquifer); 7: Boundary line of water yield property; 8: Isopleth of burial depth of aquifer group bottom plate; 9: Isopleth of thickness of aquifer in aquifer group; 10: I-I’section; 11: borehole and its numbering; 12: Silty sand; 13: Fine sand; 14: Medium sand; 15: Coarse sand; 16: Unknown particle size sand; 17: Clayey silt; 18: Mild clay; 19: Silt stratification; 20: Clay; 21: Boundary line of aquifer group; 22: The boundary of lithology in geological strata; 23: The boundary line between saltwater and freshwater (sawtooth direction is saltwater); 24: Borehole and its numbering, exposed lithology].

The study area is situated in the North China Plain, in eastern Hengshui City, Hebei Province, China, between 37°03’~37°51’N and 115°35’~116°27’E, covering an area of 3034 km². The terrain gently slopes from the southwest to the northeast, characterized by an overall flat landscape (Fig. 1). The climate of the study area belongs to the temperate monsoon climate, characterized by distinct seasons and significant temperature in humidity fluctuations. The city’s long-term average annual precipitation is 486.8 mm, with the majority of rainfall occurring between June and September. The lithology predominantly consists of limestone, dolomite, and sandstone, with carbonate rocks present in the eastern region of Gucheng County^25^. The strata are composed of Quaternary loose rock pore water, divided into four aquifer groups from top to bottom. Aquifer Groups I and II are primarily composed of medium and fine sand, containing groundwater that is SG. The upper part of Aquifer Group III is mainly medium sand with interbedded fine sand, while the lower part is dominated by fine sand with interbedded medium sand. Aquifer Group IV is composed mainly of thin to medium-thick layers of fine and coarse sand. Aquifer Groups III and IV contain groundwater that is DG (Fig. 2c). The water yield property of SG is generally low, with the specific yield of Single wells below 100 m³/(d·m) across extensive areas. In contrast, DG exhibits a higher water yield property, with the specific yield of the majority of single wells ranging between 300 and 500 m³/(d·m) (Fig. 2a, b). Deep groundwater constitutes the main water supply system for the research area.

### Data sources

Groundwater data include groundwater quality and groundwater level monitoring from 2014 to 2022. Data from 54 shallow monitoring wells in Aquifer Group I and 34 deep monitoring wells in Aquifer Group III were used (Fig. 1). Shallow wells range in depth from 4.4 m to 60 m, while deep wells range from 250 m to 385.01 m. The digital elevation model (DEM) data comes from the Geographic Spatial Data Cloud (gscloud.cn). During field sampling, the pH and total dissolved solids (TDS) of water samples were measured using a Bante902P portable multifunctional water quality analyzer. Anions (Cl⁻, SO₄²⁻, NO₃⁻, F⁻) were analyzed using Ion Chromatography (ICS-900), while cations (K⁺, Na⁺, Ca²⁺, Mg²⁺) were analyzed using Inductively Coupled Plasma Atomic Emission Spectroscopy (ICP AES-6000). CO₃²⁻ and HCO₃⁻ were determined by acid-base titration, and I⁻ was measured by the starch spectrophotometry. According to the analysis of ion balance errors as assessed by Eq. (1)^26^, the ion balance error percentages in the analyzed groundwater samples are all found to be less than 7%, that the data are within an acceptable range for ion balance, ensuring the accuracy of the groundwater analysis. In accordance with the Chinese government’s “Definition and Demarcation of Water-borne Iodine-excess Areas and Iodine-excess Endemial Areas” (GB/T19380-2016) and the “Standard for Groundwater Quality” (GB/T14848-2017), limits are set for iodine and fluoride concentration in drinking water. Groundwater is classified as high-iodine groundwater if the I⁻ concentration exceeds 0.1 mg/L and as high-fluoride groundwater if the F⁻ concentration exceeds 1 mg/L. Groundwater level data are based on annual monitoring from each well, conducted in June.

1 $$E=\frac{{\sum {{\text{z}}{{\text{m}}_{\text{c}}} - \sum {{\text{z}}{{\text{m}}_{\text{a}}}} } }}{{\sum {{\text{z}}{{\text{m}}_{\text{c}}}+\sum {{\text{z}}{{\text{m}}_{\text{a}}}} } }} \times 100$$

Where z is the absolute value of ion valence, m_c_ is the molar concentration of cationic species, m_a_ is the molar concentration of anionic species, and E represents the percentage of ion balance error.

### Research methods

To examine the temporal trends and spatial distribution of iodine and fluoride in shallow and deep groundwater from 2014 to 2022, groundwater level data and ion concentrations data (for I⁻ and F⁻) collected each June were used. Box plots showing the variation in I⁻ and F⁻ concentrations over time were generated using Origin2022, while ArcGIS10.8.1 inverse distance weighting method was employed to produce groundwater level isolines and spatial distribution diagrams. Iodine sources were analyzed using Gibbs and end-member diagrams. The Gibbs diagram classifies the main mechanisms that control the formation of chemical components in natural waters into three types: evaporation dominance, rock dominance, and precipitation dominance, based on the ratios Na⁺/(Na⁺+Ca^2+^), Cl⁻/(Cl⁻+HCO₃⁻), and TDS^27^. However, the Na⁺/(Na⁺+Ca^2+^) (or Cl⁻/(Cl⁻+HCO_2_⁻)) ratio in groundwater’s rock weathering zones exceeded the range observed in the central region of the boomerang-shaped pattern (indicating water-rock interaction), varying from 0.1 to 0.9^28^. Therefore, this study refined the Gibbs model for groundwater to expand the range of water-rock interaction considerations. Additionally, the Gibbs diagram did not adequately account for the effects of evaporation and concentration on groundwater. Since the groundwater in this study was hardly affected by evaporation and concentration, the Gibbs model can provide a preliminary analysis of the controlling factors of hydrochemistry. The end-member diagram further identifies specific types of rock weathering sources^29^. Fluoride sources were analyzed by calculating the saturation indices (SI) of dolomite, calcite, and fluorite using PHREEQC. When SI > 0, minerals are saturated (mineral precipitation); when SI < 0, minerals are unsaturated (mineral dissolution); and when SI = 0, minerals are in equilibrium^30^. The causes of iodine and fluoride evolution were analyzed using ion ratio relationships and the chloro-alkaline indices. The chloro-alkaline indices (CAI-I and CAI-II) indicate cation exchange between groundwater and the environment during its residence or migration^31^. When CAI-I and CAI-II are greater than 0, reverse cation exchange occurs, with Na⁺ and K⁺ in the groundwater exchanging with Ca^2+^ and Mg^2+^ in the aquifer. In contrast, when CAI-I and CAI-II are less than 0, positive cation exchange takes place, where Ca^2+^ and Mg^2+^ in the groundwater exchange with Na⁺ and K⁺ in the aquifer.

## Results

### Temporal distribution characteristics of iodine and fluoride

**Fig. 3:**
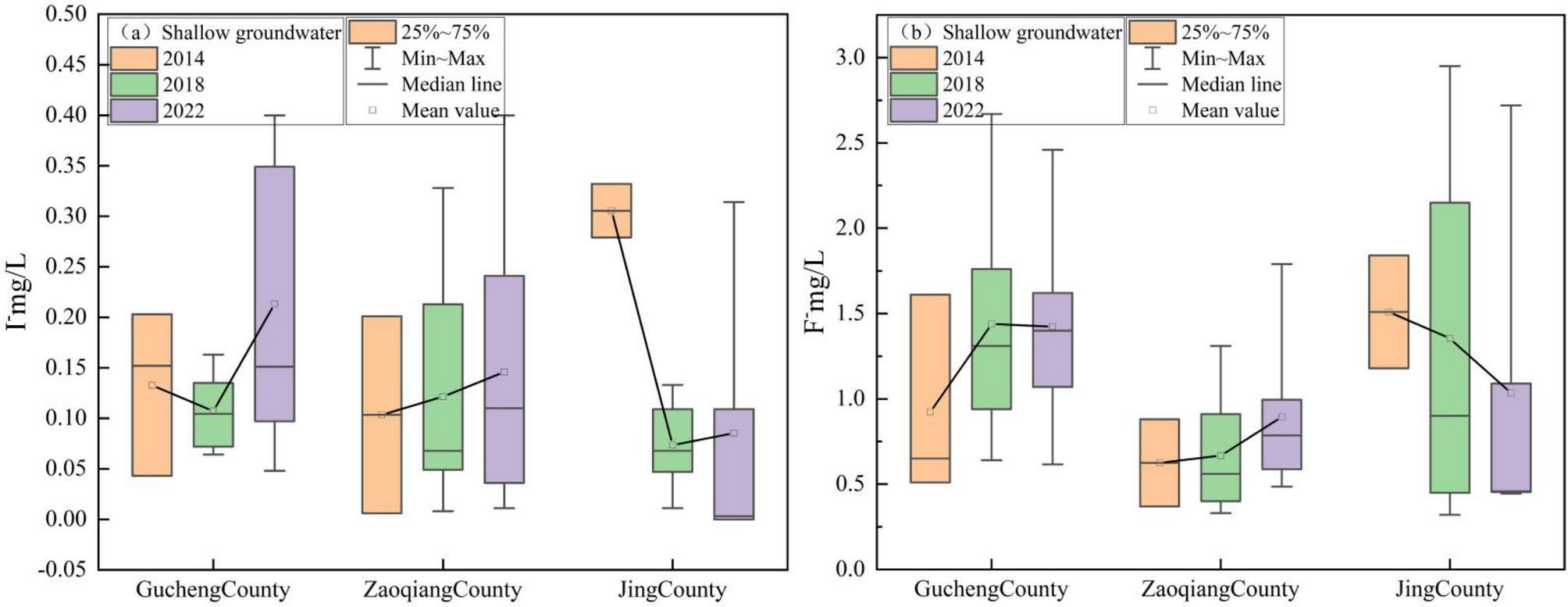
The changes in I⁻ and F⁻ concentrations in SG between 2014 and 2022.

Between 2014 and 2022, concentrations of I⁻ in the study area’s SG ranged from 0 to 0.4 mg/L, with an average that showed a decreasing trend, from 0.17 mg/L to 0.16 mg/L. Conversely, F⁻ concentrations, ranging from 0.32 to 2.95 mg/L, exhibited an increasing trend, with the average rising from 1.00 mg/L to 1.12 mg/L. In Gucheng County, the average I⁻ concentration exhibited an overall increasing trend, rising from 0.13 to 0.22 mg/L. Similarly, in Zaoqiang County, the average I⁻ concentration steadily increased from 0.10 to 0.15 mg/L. Conversely, in Jing County, the average I⁻ concentration showed a declining trend, decreasing from 0.31 to 0.08 mg/L (Fig. 3a). In Gucheng County and Zaoqiang County, the average F⁻ concentration exhibited an overall upward trend, increasing from 0.92 to 1.42 mg/L and from 0.62 to 0.89 mg/L, respectively. Conversely, in Jing County, the F⁻ concentration showed a downward trend, decreasing from 1.51 mg/L to 1.03 mg/L (Fig. 3b).

**Fig. 4:**
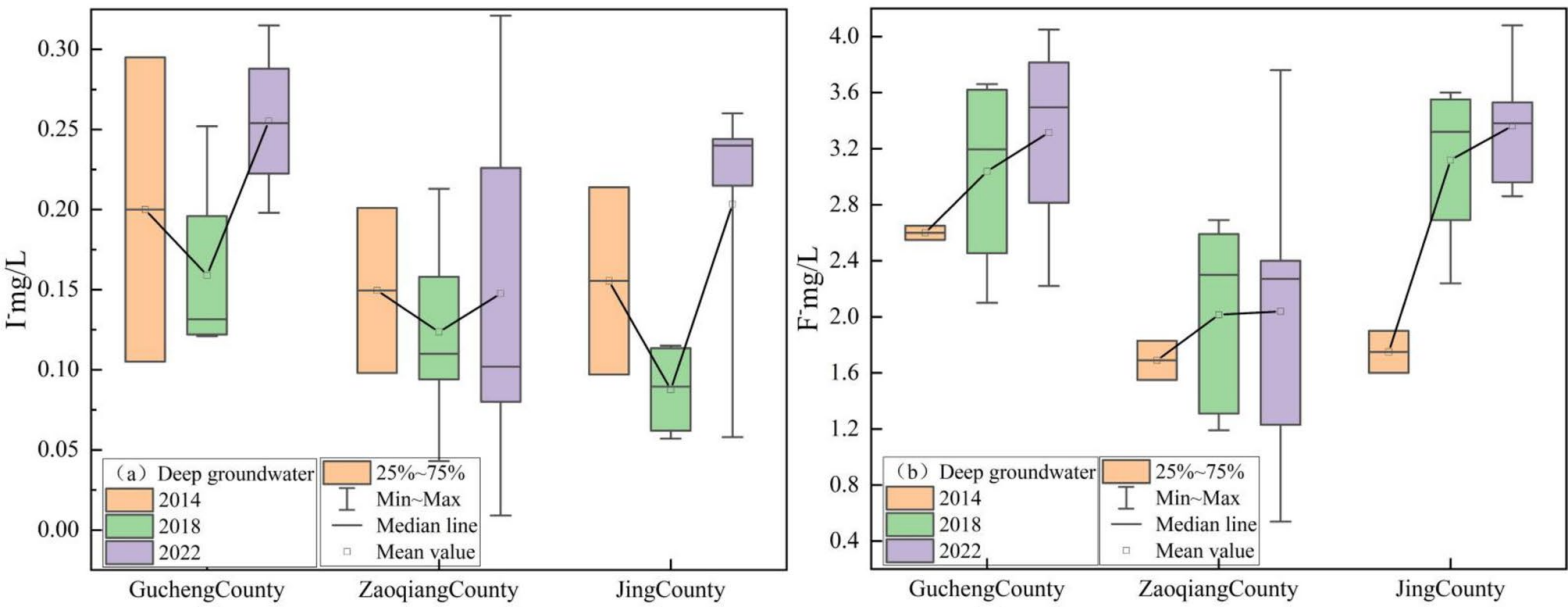
The changes in I⁻ and F⁻ concentrations in DG between 2014 and 2022.

Between 2014 and 2022, concentrations of I⁻ in the study area’s DG were observed to range between 0.01 and 0.32 mg/L, with the mean concentration increasing from 0.17 mg/L to 0.19 mg/L. Similarly, F⁻ concentrations, spanning from 0.54 to 4.08 mg/L, exhibited an upward trend, with the mean rising from 1.99 mg/L to 2.90 mg/L. In Gucheng County and Jing County, the average I⁻ concentration exhibited an overall upward trend, increasing from 0.20 to 0.26 mg/L and from 0.16 to 0.20 mg/L, respectively. In Zaoqiang County, the average I⁻ concentration remained stable, remaining stable at 0.15 mg/L both in 2014 and 2022 (Fig. 4a). In Gucheng County, Zaoqiang County, and Jing County, the average F⁻ concentration exhibited an upward trend, increasing from 2.60 to 3.32 mg/L, from 1.69 to 2.04 mg/L, and from 1.75 to 3.36 mg/L, respectively (Fig. 4b).

### Spatial distribution characteristics of iodine and fluoride

**Fig. 5:**
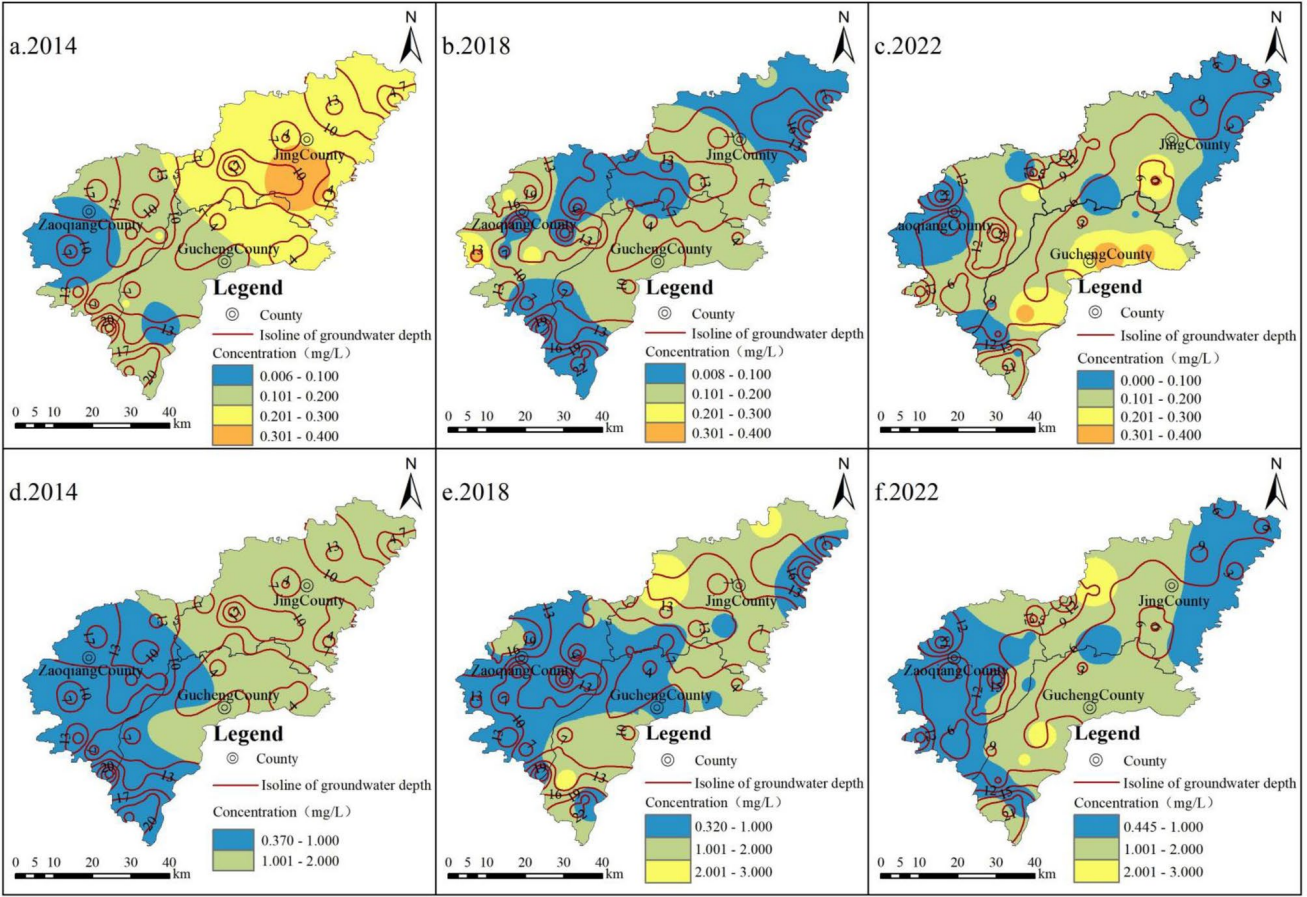
Spatial distribution of SG I⁻ and F⁻ concentrations (a-c is I⁻; d-f is F⁻) and isoline of groundwater depth from 2014 to 2022.

Between 2014 and 2022, the proportion of high-iodine groundwater in the SG study area decreased from 71.42 to 62.50%, while the proportion of high-fluoride groundwater increased from 42.86 to 45.83%. In Gucheng County, the proportion of high-iodine groundwater remained stable at 66.67%, while the proportion of high-fluoride groundwater increased from 33.33 to 77.78%. In Zaoqiang County, both the high-iodine and high-fluoride groundwater proportions rose, from 36.36 to 70.00% and from 0 to 20%, respectively. In Jing County, the proportions of high-iodine and high-fluoride groundwater both declined, decreasing from 100% in 2014 to 40% in 2022 (Fig. 5). From the perspective of spatial distribution, groundwater flow influences ion migration. The highest water levels are observed in the recharge areas located in the southwest of Gucheng County and Zaoqiang County, while the lowest water levels occur in the discharge area in the northeast of Jing County. Overall, the groundwater flows from southwest to northeast. I⁻ and F⁻ concentrations were lower in the recharge area compared to the discharge area, most significantly in 2014. This was attributed to the shorter hydraulic residence time and flow paths in the recharge area. However, with long-term management of groundwater exploitation reduction, this pattern has gradually diminished. I⁻ and F⁻ concentrations in the recharge area have steadily increased, while concentration in the discharge area have gradually declined (Fig. 5).

**Fig. 6:**
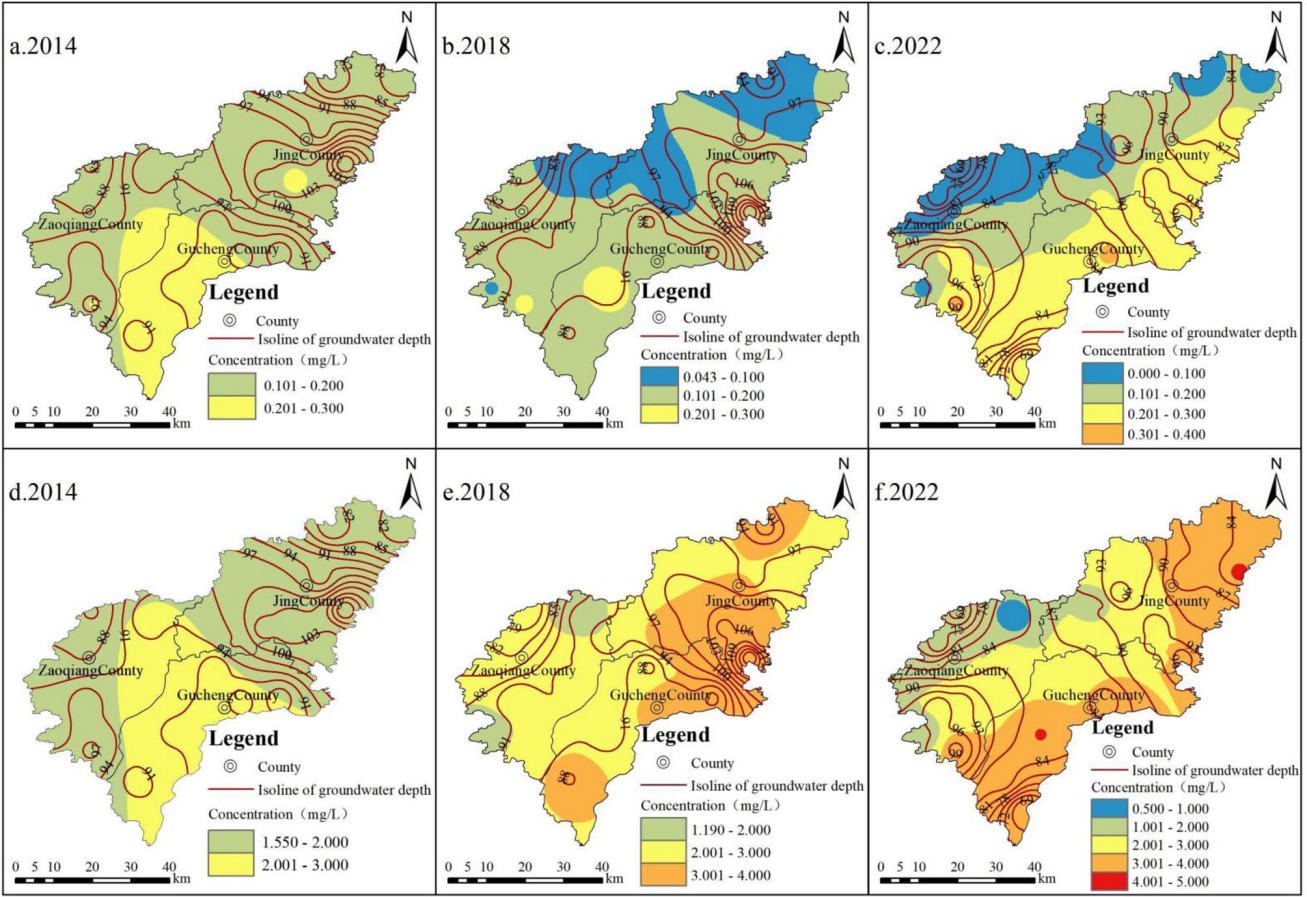
Spatial distribution of DG I⁻ and F⁻ concentrations (a-c is I⁻; d-f is F⁻) and isoline of groundwater depth from 2014 to 2022.

Between 2014 and 2022, the proportion of high-iodine groundwater in the DG of the study area decreased from 100 to 66.67%, and the proportion of high-fluoride groundwater decreased from 100 to 93.33%. In Gucheng County, the proportions of high-iodine and high-fluoride groundwater remained constant at 100%. In Zaoqiang County, the proportions of high-iodine and high-fluoride groundwater both declined, from 100 to 60% and from 100 to 80%, respectively. In Jing County, the proportion of high-iodine groundwater dropped from 100 to 66.67%, while high-fluoride groundwater remained stable at 100% (Fig. 6). In the DG, the recharge areas are located in the northwest of Zaoqiang County and Jing County, where water levels are higher. The discharge areas, with lower water levels, are found in the southeast of Gucheng County and Jing County. Groundwater flows from the northwest to the southeast. The spatial distribution of I⁻ and F⁻ concentrations in deep groundwater is nearly identical, with ion concentrations increasing progressively along the groundwater flow direction. I⁻ and F⁻ concentrations are lower in the recharge areas compared to the discharge areas (Fig. 6).

### Main sources of iodine in groundwater

**Fig. 7:**
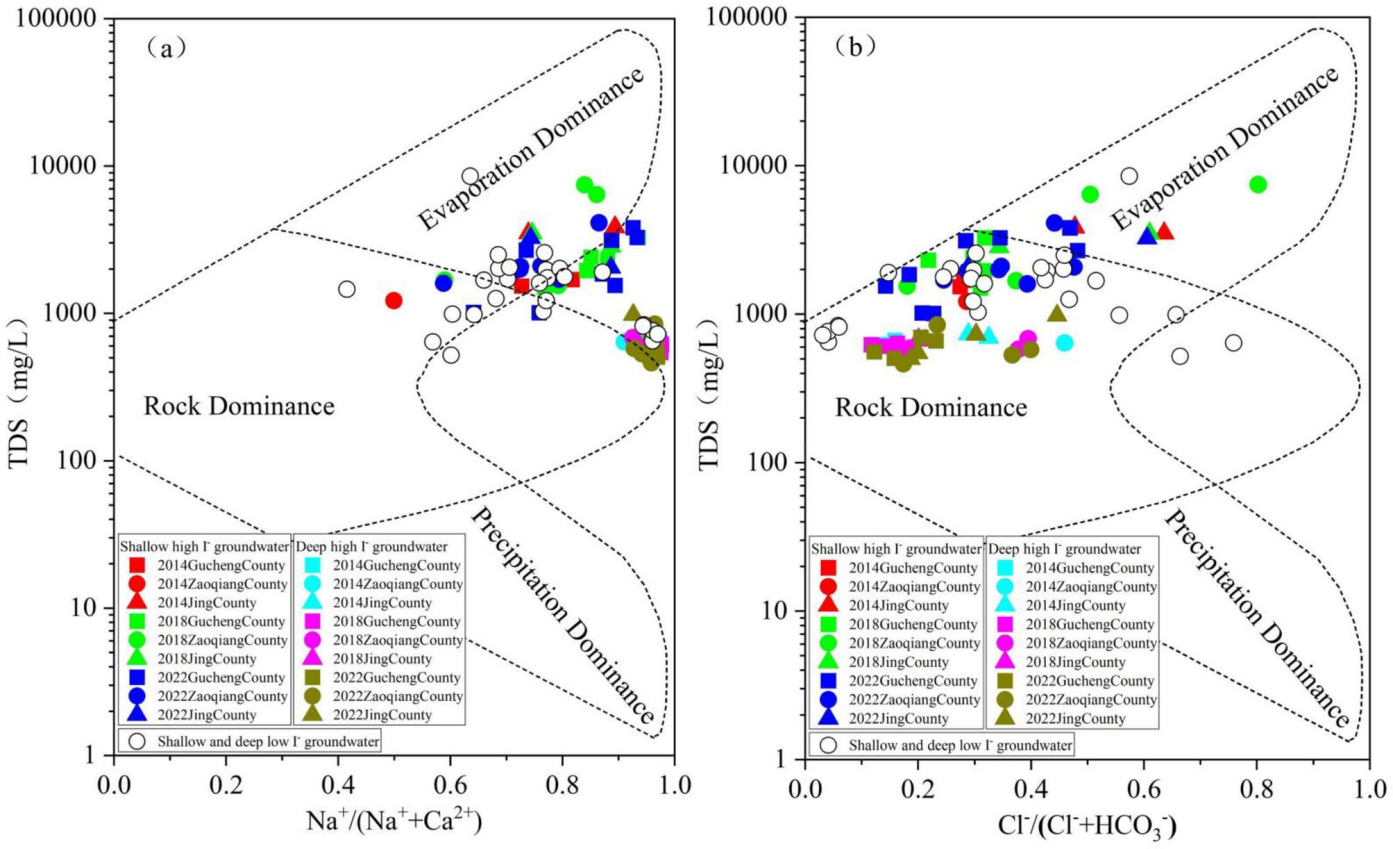
Between 2014 and 2022, gibbs diagram (**a**,**b**) for SG and DG.

Between 2014 and 2022, the Na⁺/(Na⁺+Ca²⁺) values in SG ranged from 0.42 to 0.93, with an average of 0.76, the Cl⁻/(Cl⁻+HCO₃⁻) values ranged from 0.14 to 0.80, with an average of 0.36, while TDS values ranged from 642.00 to 8541.30 mg/L, with an average of 2363.69 mg/L. SG quality sampling points were mainly distributed in rock and evaporation dominance areas. In DG, the Na⁺/(Na⁺+Ca^2+^) values ranged from 0.60 to 0.98, with an average of 0.94, the Cl⁻/(Cl⁻+HCO₃⁻) values ranged from 0.12 to 0.46, with an average of 0.26, and TDS values ranged from 462.00 to 982.00 mg/L, with an average of 648.71 mg/L. DG quality sampling points were mainly distributed in rock dominance area (Fig. 7). The results suggest that high-iodine groundwater in SG is primarily influenced by evaporation concentration and rock weathering, whereas in DG, it is mainly affected by rock weathering, In the Na⁺/(Na⁺+Ca²⁺) plot for SG and DG, the sampling points are concentrated near the edge of the water-rock interaction model box, indicating that both SG and DG are also influenced by other factors.

**Fig. 8:**
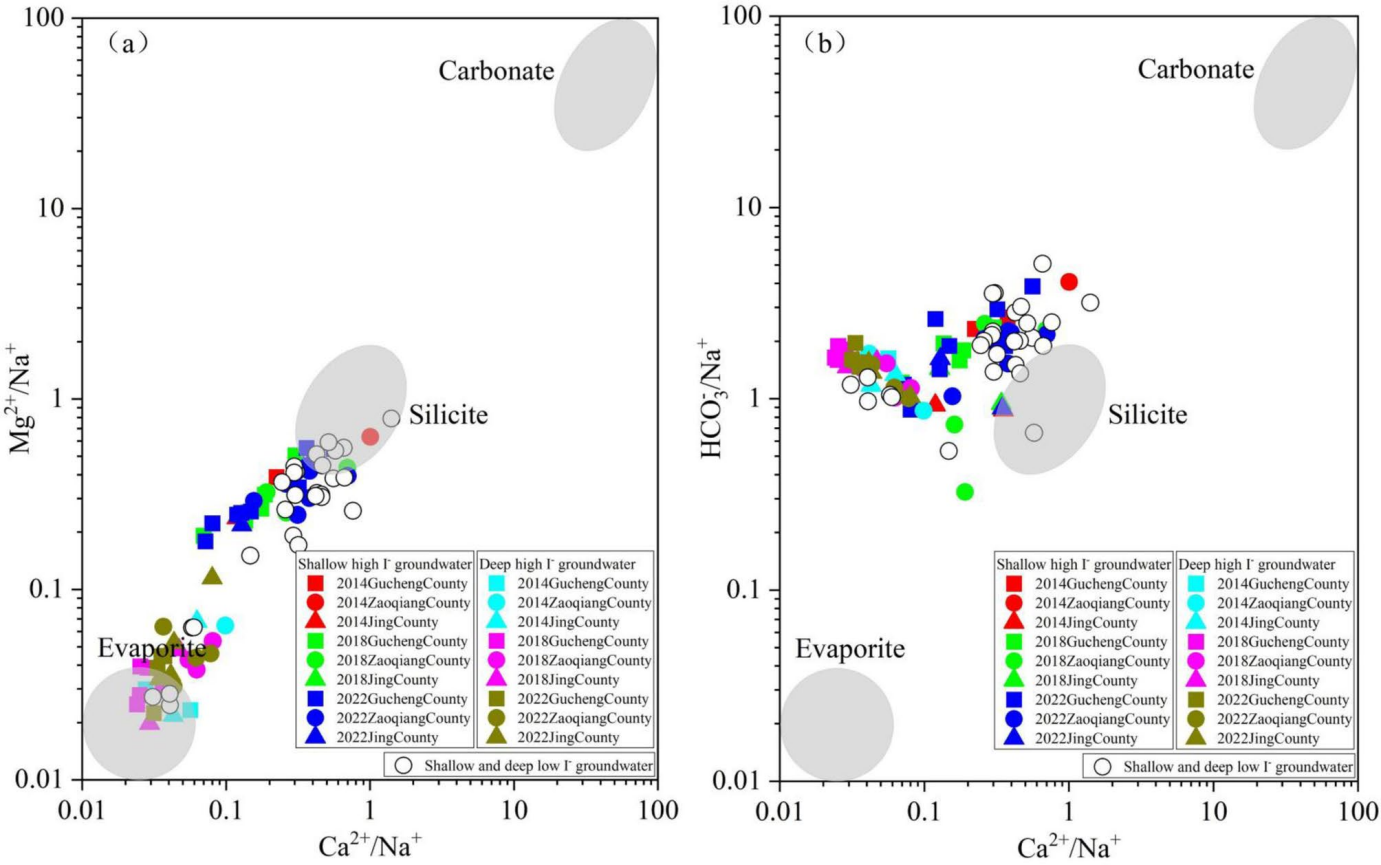
Between 2014 and 2022, end-member diagram (**a**,**b**) for SG and DG.

To further identify the types of rock weathering sources associated with the hydrochemical characteristics of groundwater in the study area, a Mg/Na⁺, HCO₃⁻/Na⁺, and Ca^2+^/Na⁺ end-member diagram was developed. Between 2014 and 2022, groundwater quality sampling points from both SG and DG were predominantly located near the silicate and evaporite areas, distant from the carbonate area (Fig. 8). The results indicate that between 2014 and 2022, the hydrochemical composition of high-iodine groundwater was primarily derived from silicate and evaporite weathering, and is virtually unaffected by carbonate dissolution processes.

### Main sources of fluoride in groundwater

**Fig. 9:**
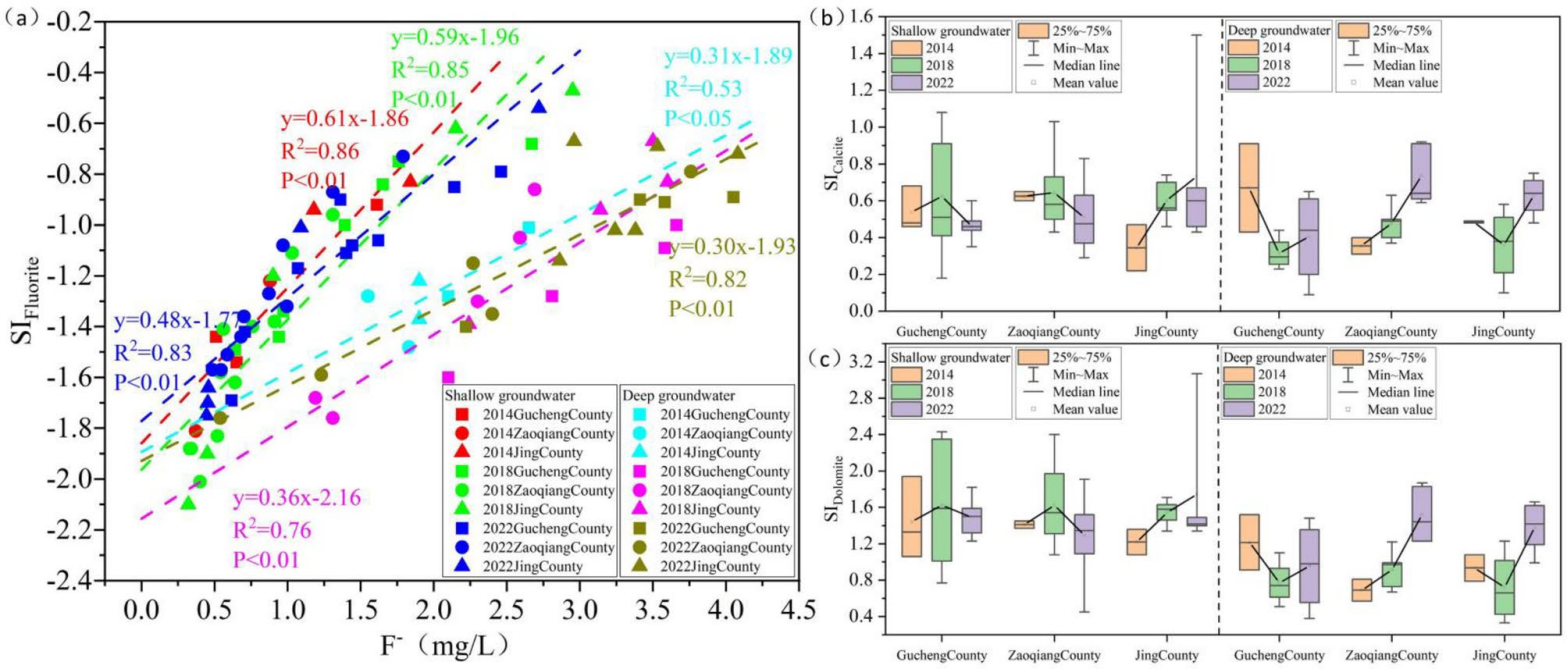
Between 2014 and 2022, the relationship between F⁻ concentration and the fluorite saturation index (a), with the saturation indices of calcite (b) and dolomite (c).

Mineral dissolution and precipitation are the main processes involved in water-rock interactions. The dolomite and calcite mineral saturation indices in both SG and DG are greater than 0, and the fluorite mineral saturation index is less than 0. The results indicate that between 2014 and 2022, dolomite and calcite in both SG and DG are in a precipitated state, whereas fluorite is in a dissolved state. In SG and DG, F⁻ is significantly correlated with the fluorite saturation index (Fig. 9a). Based on the fluorite dissolution Eq. (2), the results indicate that between 2014 and 2022, F⁻ in both SG and DG primarily originated from the dissolution of fluorite minerals^32,33^.

2 $$Ca{F_2}=C{a^{2+}}+2{F^ - }$$

Dolomite and calcite precipitation, as shown in Eqs. (3) and (4), reduces Ca²⁺ concentration in groundwater. This decrease promotes the dissolution of fluorite, resulting in an increase in F⁻ concentration^34^.

3 $$C{a^{2+}}+HCO_{{_{3}}}^{ - } \to CaC{O_3}+C{O_2}+{H_2}O~$$

4 $$C{a^{2+}}~+{\text{ }}M{g^{2+}}+2HCO_{3}^{ - }+2O{H^ - } \to CaMg{\left( {C{O_3}} \right)_2}+{\text{ }}{H_2}O$$

Between 2014 and 2022, the saturation indices for calcite and dolomite in SG in Gucheng County remained stable. In Zaoqiang County, the calcite saturation index showed a decreasing trend, while the dolomite saturation index remained stable. In Jing County, both the calcite and dolomite saturation indices increased (Fig. 9b, c). The findings indicate that from 2014 to 2022, calcite and dolomite precipitation in SG consistently promoted F⁻ concentration in Gucheng County, had a slightly diminished effect in Zaoqiang County, and showed an increasing effect in Jing County. The stronger effect in Jing County is likely due to its location in a discharge area, where evaporation concentration processes favor mineral precipitation (Fig. 7).

Between 2014 and 2022, the calcite and dolomite saturation indices in DG in Gucheng County decreased, whereas in Zaoqiang County and Jing County, both indices increased (Fig. 9b, c). The findings indicate that the precipitation of calcite and dolomite in DG between 2014 and 2022 has weakened the promotion of F⁻ concentration in Gucheng County, while the promotion in Zaoqiang County and Jing County has gradually increased.

## Discussion

### Groundwater hydraulic conditions

Groundwater hydraulic conditions play a key role in the migration and accumulation of ions in groundwater. The Ca²⁺/Cl⁻ ratio is indicative of groundwater hydraulic conditions; lower values suggest slower flow velocities and smaller groundwater fluctuations^35^.

#### Shallow groundwater hydraulic conditions

**Fig. 10:**
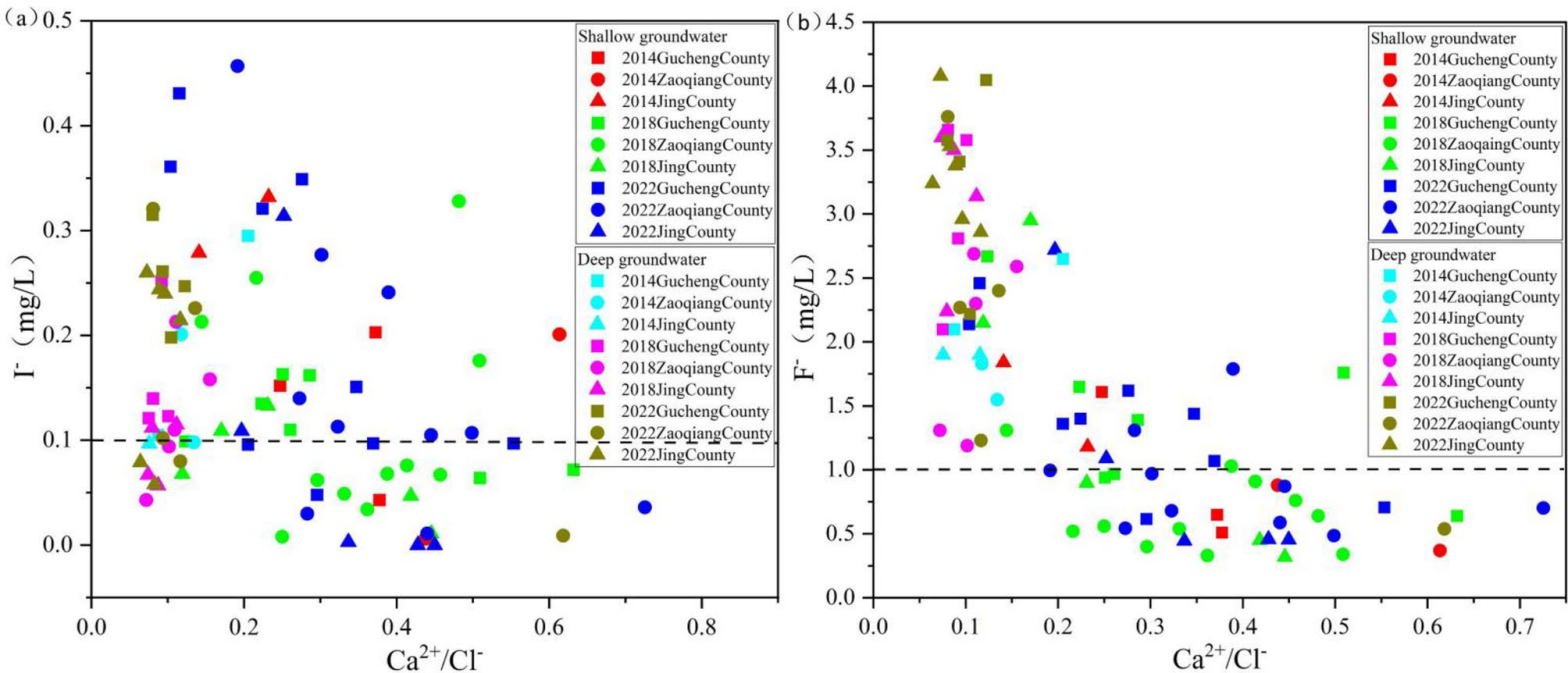
Relationship between I⁻ and F⁻ concentrations and the Ca²⁺/Cl⁻ ratio in SG and DG from 2014 to 2022 (high-iodine and high-fluoride groundwater sampling points above the dashed line).

Significant changes in groundwater hydraulic conditions were observed in SG during the groundwater exploitation reduction control period. Compared to 2014, groundwater hydraulic conditions deteriorated in 2022 in Gucheng County and Zaoqiang County (mean values decreased from 0.33 to 0.28 and from 0.53 to 0.39, respectively), while the opposite was true in Jing County (mean value increased from 0.19 to 0.33), with Jing County having the poorest groundwater hydraulic conditions in 2014 (mean value of 0.19) (Fig. 10). Poor groundwater hydraulic conditions slow the renewal process, leading to the gradual accumulation of I⁻ and F⁻ concentrations during groundwater movement, which results in high-iodine and high-fluoride groundwater. Combined with spatial distribution (Fig. 5), SG groundwater hydraulic conditions gradually weakened along the flow direction in 2014. In contrast, by 2022, these conditions have gradually improved along the same flow path. In the discharge area, water levels are low, forming a funnel area center. During the period of groundwater over-exploitation, the groundwater level dropped significantly, creating a depression funnel. This decline increased the distance between groundwater and the surface, reducing natural recharge from surface water. It may have also altered groundwater flow paths, making them more complex or weaker, leading to the poorest groundwater flow conditions in 2014. During the control period, the strong disturbances to groundwater caused by over-exploitation and artificial recharge improved groundwater hydraulic conditions in Jing County. Intense groundwater exchange may have migrated and diluted I⁻ and F⁻, leading to a reduction in their concentrations in the groundwater of Jing County.

#### Deep groundwater hydraulic conditions

The range of DG Ca²⁺/Cl⁻ ratio is more concentrated and less variable. By 2022, groundwater hydraulic conditions in Gucheng County, Zaoqiang County, and Jing County had slightly weakened compared to 2014, though they remained largely stable. The average values declined from 0.15 to 0.09 in Gucheng County, from 0.12 to 0.11 in Zaoqiang County, and 0.10 to 0.09 in Jing County (Fig. 10). During the comprehensive management of groundwater over-exploitation, the intensity of deep groundwater flow decreased. Combined with the spatial distribution (Fig. 6), groundwater hydraulic conditions in the recharge area were stronger than in the discharge area, resulting in the co-enrichment of iodine and fluoride in the discharge area.

### Hydrochemical conditions and redox environment

**Fig. 11:**
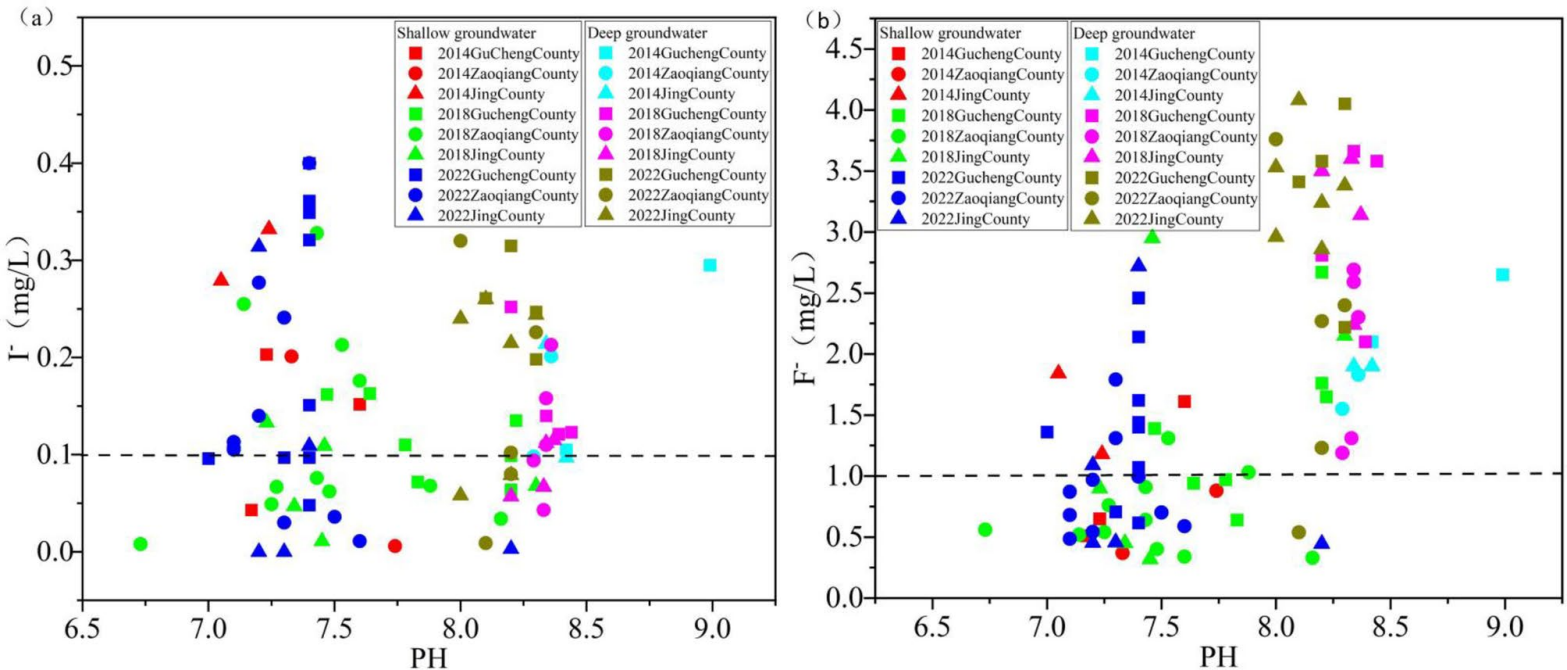
Relationship between I⁻ and F⁻ concentrations and pH in SG and DG from 2014 to 2022 (high-iodine and high-fluoride groundwater sampling points above the dashed line).

pH plays a crucial role in influencing the enrichment of iodine and fluoride^36,37^. The pH of SG predominantly ranges from 7.00 to 7.50, while that of DG ranges from 8.00 to 8.50 (Fig. 11). This indicates that the groundwater in the study area is alkaline, which favors the accumulation of I⁻ and F⁻.

#### Shallow groundwater redox environment

The SO₄²⁻/(HCO₃⁻+CO₃²⁻) ratio serves as an indicator for assessing the redox environment of groundwater^38^. Lower ratios values indicate a stronger reducing environment in the groundwater. A reducing environment promotes the accumulation of I⁻ and F⁻ in groundwater^39^.

**Fig. 12:**
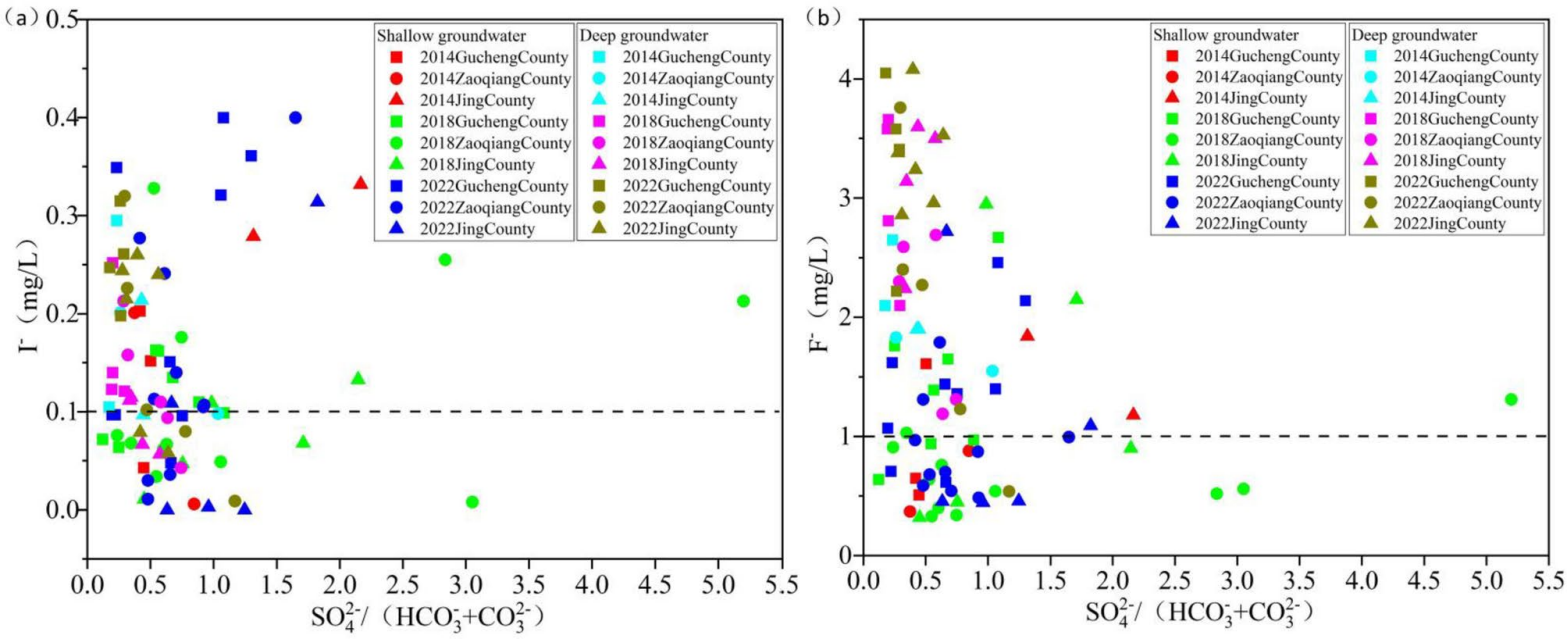
Relationship between I⁻ and F⁻ concentrations and SO₄²⁻/(HCO₃⁻+CO₃²⁻) ratio in SG and DG from 2014 to 2022 (high-iodine and high-fluoride groundwater sampling points above the dashed line).

Between 2014 and 2022, the average SO₄²⁻/(HCO₃⁻+CO₃²⁻) ratios in SG increased from 0.45 to 0.68 in Gucheng County and from 0.61 to 0.74 in Zaoqiang County. In contrast, a decrease was observed in Jing County, where the ratio decreased from 1.74 to 1.06 (Fig. 12). Compared to Gucheng County and Zaoqiang County, Jing County exhibited significant changes in the intensity of their reducing environment. However, Gucheng County and Zaoqiang County consistently maintained a stronger reducing environment. Combined with spatial distribution (Fig. 5), the reducing environment in SG weakens progressively from the recharge area to the discharge area along the groundwater flow path. Since Jing County is situated at the center of the groundwater funnel area, the groundwater hydraulic conditions there are stronger compared to Gucheng County and Zaoqiang County (Fig. 10). Frequent interactions between groundwater and the atmosphere have led to consistently weakest reducing environment in Jing County compared to Gucheng County and Zaoqiang County.

#### Deep groundwater redox environment

Between 2014 and 2022, the mean values of SO₄²⁻/(HCO₃⁻+CO₃²⁻) in DG in Gucheng County, Zaoqiang County, and Jing County ranged from 0.20 to 0.25, 0.61 to 0.65, and 0.42 to 0.44, respectively. While minor fluctuations were observed, the mean values remained generally stable (Fig. 12). Of the three counties, Gucheng County is most influenced by the reducing environment, whereas Zaoqiang County is the least affected. Combined with spatial distribution (Fig. 6), the reducing environment in DG progressively intensifies along the flow path from the recharge area to the discharge area. This is primarily due to the flow of DG within a closed, slow-velocity environment. In the recharge area, oxidizing environments are relatively favorable. However, as groundwater moves slowly, oxygen is gradually depleted, resulting in the enhancement of reducing environment.

#### Relationship between I⁻ and F⁻ with HCO₃⁻ and Ca²⁺ in shallow groundwater

The changes in HCO₃⁻ and Ca²⁺ concentrations in groundwater may be affected by artificial recharge^40^. During artificial recharge, the groundwater environment undergoes changes. Before artificial recharge, Ca²⁺ primarily originates from the dissolution of plagioclase, while Mg²⁺ is mainly derived from the dissolution of dolomite. After artificial recharge, HCO₃⁻ and Ca²⁺ are predominantly sourced from the dissolution of calcite and dolomite^41^. A competitive adsorption relationship exists between HCO₃⁻ and I⁻ in groundwater, and when the concentration of HCO₃⁻ in groundwater is high, the concentration of I⁻ correspondingly increases^42^. This mechanism is primarily evident in alkaline environments. Not only I⁻ is affected by HCO₃⁻, but F⁻ is likewise affected by HCO₃⁻. The reaction equation of (5) shows that when fluorite is dissolved in groundwater with high HCO₃⁻ content, HCO₃⁻ type water, which is alkaline and has a low Ca²⁺ concentration, promotes fluorite dissolution, leading to an increase in F⁻ concentration^43^.

5 $$Ca{F_2}+2HCO_{3}^{ - }+O{H^ - }=CaC{O_3}+2{F^ - }+{H_2}O+C{O_2}$$

**Fig. 13:**
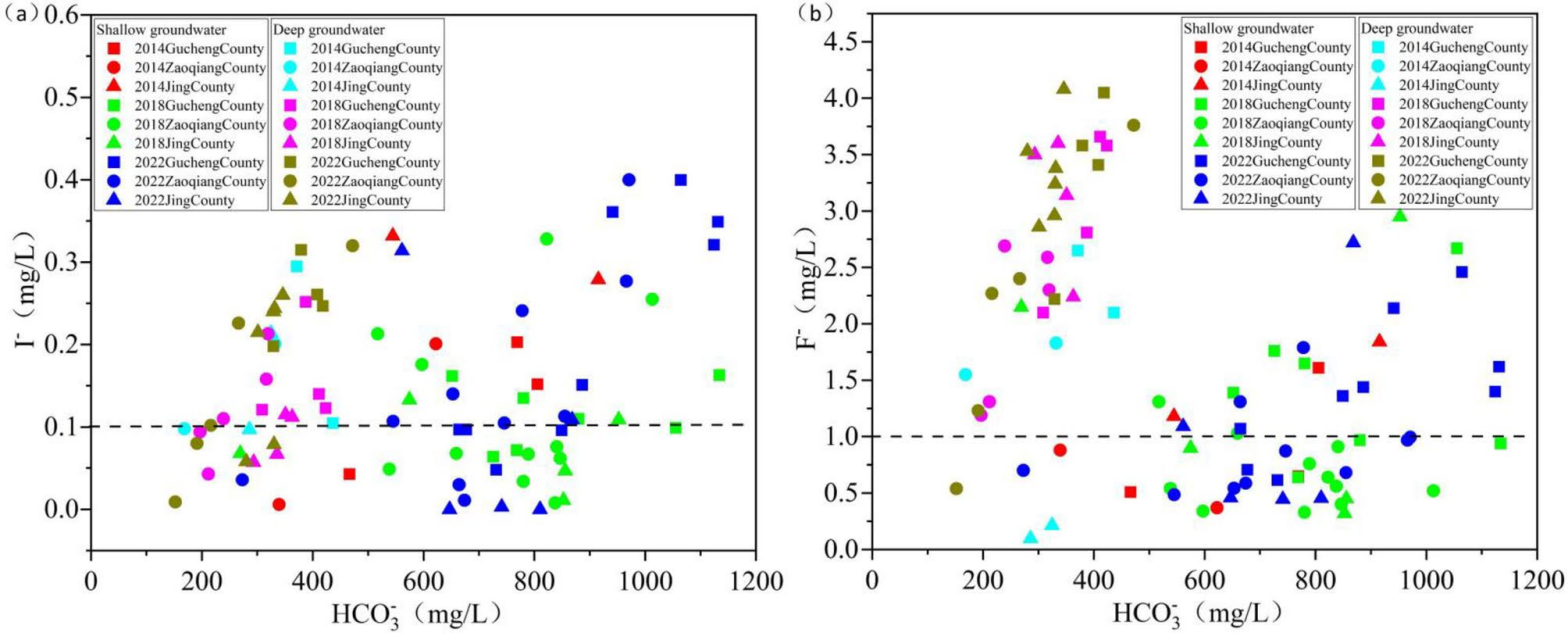
Relationship between I⁻ and F⁻ concentrations and HCO₃⁻ concentration in SG and DG from 2014 to 2022 (high-iodine and high-fluoride groundwater sampling points above the dashed line).

Between 2014 and 2022, the average HCO₃⁻ concentration in SG rose from 680.13 mg/L to 896.33 mg/L in Gucheng County and from 480.85 mg/L to 712.50 mg/L in Zaoqiang County. In contrast, in Jing County, the average concentration decreased slightly from 729.80 mg/L to 725.40 mg/L. The HCO₃⁻ concentrations in Gucheng County and Zaoqiang County exhibited a significant increase, whereas in Jing County, the concentration remained relatively stable (Fig. 13). These findings suggest that the influence of HCO₃⁻ concentration on I⁻ and F⁻ concentration has gradually strengthened in Gucheng County and Zaoqiang County, whereas in Jing County, it has remained relatively constant.

**Fig. 14:**
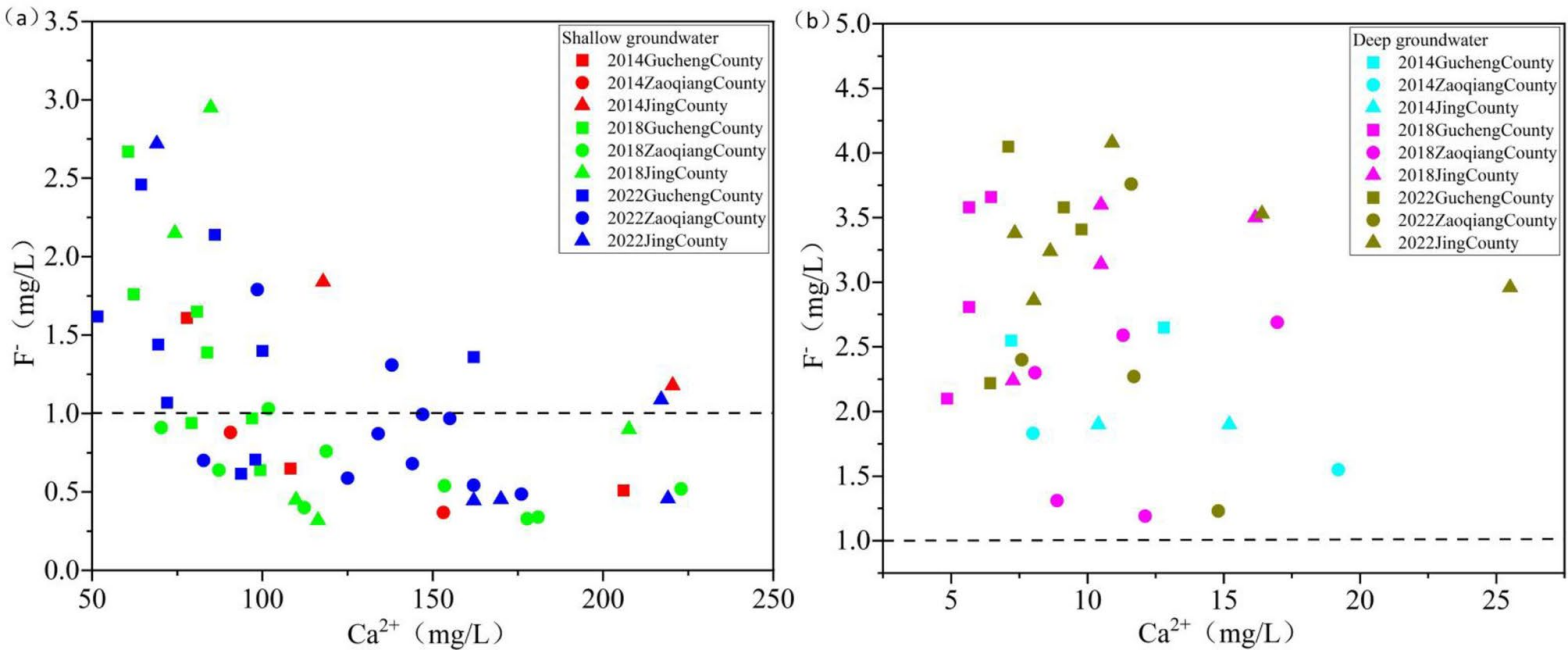
Relationship between I⁻ and F⁻ concentrations and Ca²⁺ concentration in SG and DG from 2014 to 2022 (high-iodine and high-fluoride groundwater sampling points above the dashed line).

Between 2014 and 2022, the average Ca²⁺ concentration in SG in Gucheng County decreased from 130.67 mg/L to 88.56 mg/L. In Zaoqiang County, it increased from 121.85 mg/L to 136.22 mg/L, while in Jing County, it remained nearly unchanged, with a minor decrease from 169.1 mg/L to 167.40 mg/L (Fig. 14a). It is indicated that in Gucheng County, the F⁻ concentration was promoted by the increase in Ca²⁺ concentration, while in Zaoqiang County, the influence of Ca²⁺ on F⁻ concentration diminished. In Jing County, this relationship remained nearly unchanged.

#### Relationship between I⁻ and F⁻ with HCO₃⁻ and Ca²⁺ in deep groundwater

Between 2014 and 2022, the average HCO₃⁻ concentration in DG in Gucheng County decreased from 403.60 mg/L to 383.50 mg/L. In Zaoqiang County, it increased from 250.15 mg/L to 259.40 mg/L, while in Jing County, it rose from 305.10 mg/L to 319.50 mg/L. The HCO₃⁻ concentrations in DG were generally lower and exhibited less variation compared to SG (Fig. 13). It is indicated that from 2014 to 2022, the concentrations of I⁻ and F⁻ in DG remained largely stable and were not significantly influenced by variations in HCO₃⁻ concentration.

Between 2014 and 2022, the average Ca²⁺ concentration in DG in Gucheng County decreased from 10.00 mg/L to 8.11 mg/L. In Zaoqiang County, it increased from 13.6 mg/L to 19.80 mg/L, while in Jing County, it remained nearly constant at 12.8 mg/L (Fig. 14b). It is indicated that in Gucheng County, the influence of Ca²⁺ on F⁻ concentration increased, while in Zaoqiang County, this effect diminished. In Jing County, the relationship remained nearly unchanged.

### Ion exchange

**Fig. 15:**
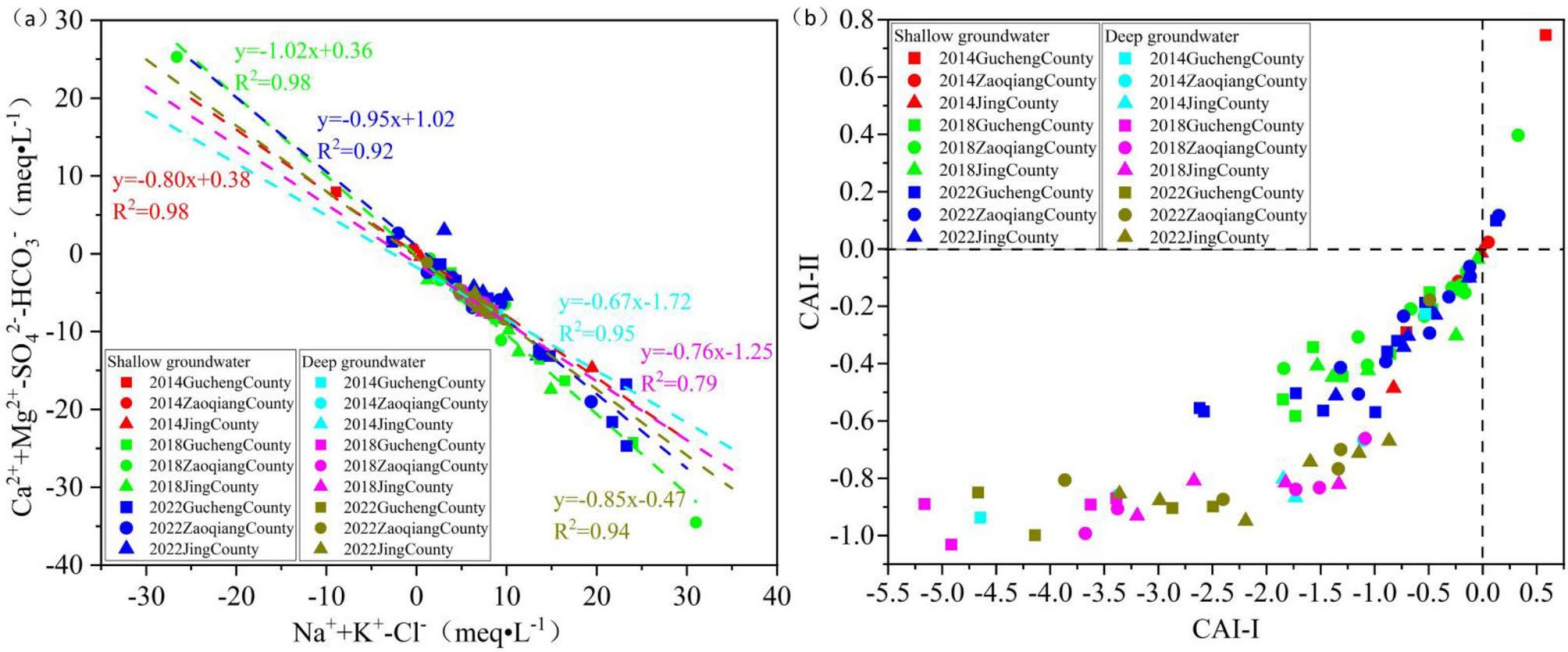
Cation exchange (a) and chloro-alkaline indices diagram (b) for SG and DG between 2014 and 2022.

Cation exchange in groundwater is indicated when the ratio (Ca²⁺+Mg²⁺-SO₄²⁻-HCO₃⁻)/(Na⁺+K⁺-Cl⁻) approaches − 1^14^. Between 2014 and 2022, the fitted linear equations for SG sampling points were y=-0.80x + 0.38, R²=0.98 ; y=-1.02x + 0.36, R²=0.98 ; y=-0.95x + 1.02, R²=0.92. For DG sampling points, the fitted linear equations were y=-0.67x-1.72, R²=0.95 ; y=-0.76x-1.25, R²=0.79 ; y=-0.85x-0.47, R²=0.94 (Fig. 15a). It is indicated that cation exchange is weaker in DG and stronger in SG. This is due to the relatively flat terrain of the study area, the slow flow of SG, and the fine-grained nature of the aquifer sediments, cation exchange is more pronounced. Compared to 2014, cation exchange had been enhanced in both SG and DG by 2022.The chloro-alkaline indices were used to further analyze the strength and direction of cation exchange in groundwater.

#### Shallow groundwater cation exchange

Between 2014 and 2022, the majority of SG sampling points had CAI-I < 0 and CAI-II < 0, indicating positive cation exchange at most locations, with the exchange being stronger in 2022 compared to 2014. Some sampling points exhibit CAI-I > 0 and CAI-II > 0, reverse cation exchange occurs. This is because salt water is distributed in the SG (Fig. 2c), where the high concentrations of Na⁺ and K⁺ in the salt water displace Ca²⁺ and Mg²⁺ from the aquifer minerals. This leads to the formation of high Ca²⁺, which in turn inhibits the enrichment of F⁻.

#### Deep groundwater cation exchange

At all DG sampling points, CAI-I < 0 and CAI-II < 0, indicating the occurrence of positive cation exchange. Compared to SG, the CAI-I and CAI-II values in DG are more concentrated and have lower averages, indicating stronger positive cation exchange in DG (Fig. 15b). The aquifer in the study area is composed of fine particles with a large specific surface area. GER has reduced the flow velocity (Fig. 10), extended retention time and creating favorable conditions for cation exchange adsorption. This leads to high TDS water dominated by Na⁺ and Cl⁻, facilitating I⁻ enrichment^44^. Ca²⁺ is more readily adsorbed onto particle surfaces than Na⁺, causing Na⁺ that is adsorbed on mineral surfaces to exchange with Ca²⁺ in groundwater. This process creates a groundwater environment high in Na⁺ and low in Ca²⁺, which promotes F⁻. enrichment^45^.

### Human activities

**Fig. 16:**
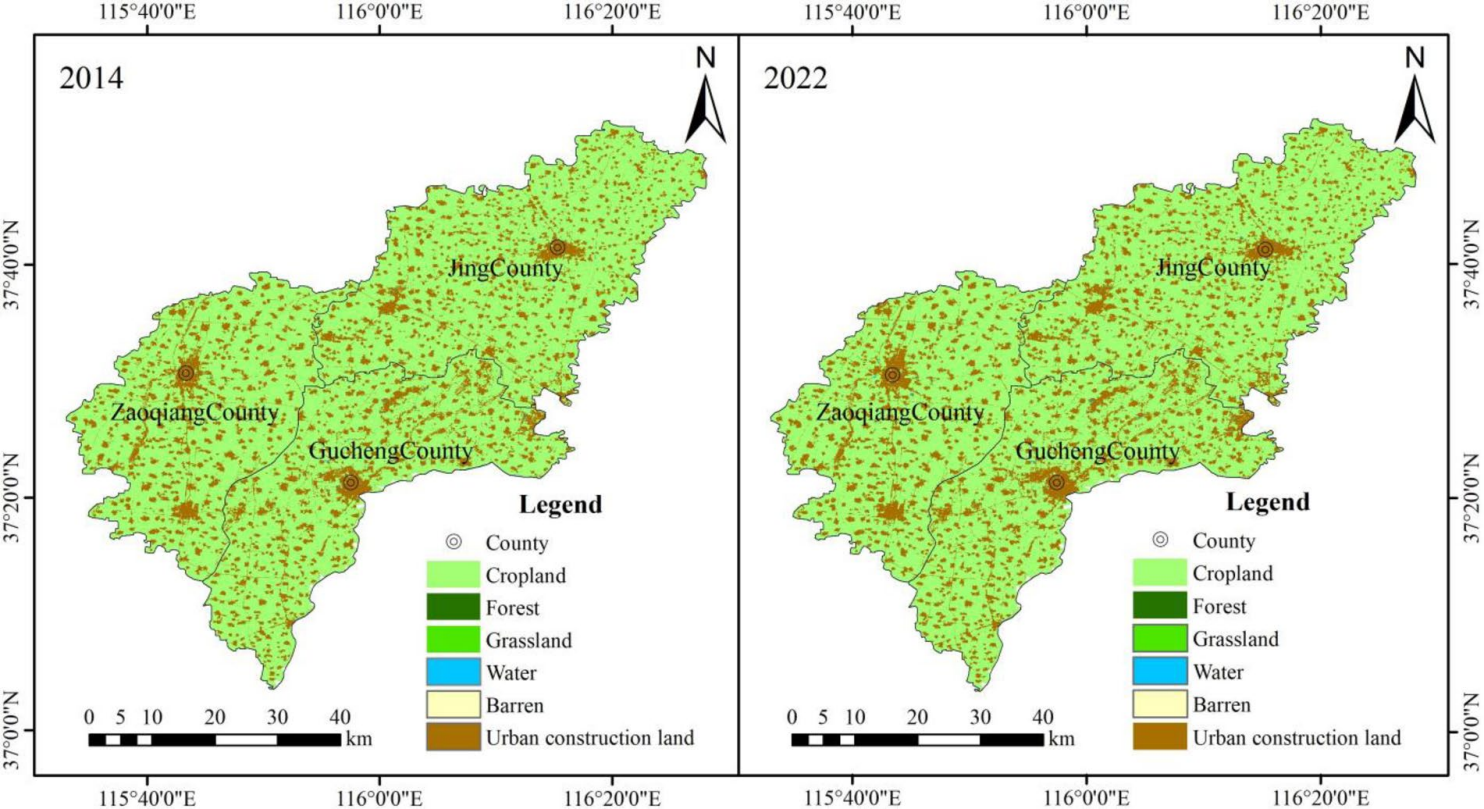
Land use types in the study area for the years 2014 and 2022.

Among the land use types in the study area, farmland occupies the largest area (Fig. 16). In recent decades, wheat and corn have been the primary crops cultivated in the area, accounting for a significant proportion of the cultivation^46^. Agricultural irrigation during cultivation modifies the groundwater hydraulic conditions, causing the translocation of I⁻ and F⁻ concentrations. The substantial application of fertilizers, rich in magnesium sulfate, sodium nitrate, calcium oxide, and potassium chloride, has contributed to the rise in concentrations of Na⁺, Mg²⁺, Ca²⁺, K⁺, and Cl⁻. Consequently, this has led to an increase in TDS levels within the groundwater^14^, subsequently enhancing the concentrations of I⁻ and F⁻.

## Conclusion

The analysis of the evolution characteristics and causes of iodine and fluoride in groundwater in the eastern funnel area of Hengshui City during the GER period has led to the following conclusions:

1) Between 2014 and 2022, the I⁻ concentration in the study area’s SG ranged from 0 to 0.4 mg/L, and the F⁻ concentration ranged from 0.32 to 2.95 mg/L. In Gucheng County and Zaoqiang County, the average concentrations of I⁻ and F⁻ increased, while in Jing County, they decreased. In DG, the I⁻ concentration ranged from 0.01 to 0.32 mg/L, and F⁻ concentration ranged from 0.54 to 4.08 mg/L. The average I⁻ concentration increased in Gucheng and Jing counties but remained stable in Zaoqiang County. Meanwhile, the average F⁻ concentration increased in Gucheng County, Zaoqiang County, and Jing County.

2) From 2014 to 2022, the proportion of high-iodine groundwater in the SG of the study area had decreased from 71.42 to 62.50%, while the proportion of high-fluoride groundwater had increased from 42.86 to 45.83%. In DG, the proportion of high-iodine groundwater decreased from 100 to 66.67%, while high-fluoride groundwater decreased from 100 to 93.33%.

3) In 2014, the concentrations of I⁻ and F⁻ in SG increased progressively from the recharge area to the discharge area along the groundwater flow path. In contrast, in 2018 and 2022, the I⁻ and F⁻ concentrations increased in the recharge area but decreased in the discharge area. Between 2014 and 2022, the concentrations of I⁻ and F⁻ in DG progressively increased from the recharge area to the discharge area along the groundwater flow path.

4) Before and after GER, the primary sources of I⁻ and F⁻ in both SG and DG remained consistent. Iodine in SG was mainly derived from evaporation concentration, silicate, and evaporite weathering processes, while in DG, it primarily originated from silicate and evaporite weathering, and is virtually unaffected by carbonate dissolution. Fluoride in SG and DG is primarily derived from the dissolution of fluorite minerals. Nevertheless, in the context of prolonged GER and recharge, the concentrations of I⁻ and F⁻ have undergone changes. The evolution of I⁻ and F⁻ concentrations in SG is primarily influenced by the influenced by groundwater hydraulic conditions and high HCO₃⁻ concentration. The concentrations of I⁻ and F⁻ in DG are primarily influenced by the facilitating effect of reducing environment, low Ca²⁺ concentration, and positive cation exchange. In addition, human activities may also influence the enrichment of I⁻ and F⁻ concentrations in groundwater through the seepage of various ions from agricultural irrigation and fertilizers.

## Electronic supplementary material

Below is the link to the electronic supplementary material.

Supplementary Material 1

## Data Availability

The datasets used and/or analysed during the current study available from the corresponding author on reasonable request.
