# Supplementary material for: Evolution characteristics and causes of iodine and fluoride in groundwater of Hengshui city in North China
Source: Sci Rep. 2024 Dec 30;14:32052. doi: 10.1038/s41598-024-83601-2 (PMC11685976; doi:10.1038/s41598-024-83601-2)
Supplement: Supplementary file 1 — Supplementary Material 1 [file 41598_2024_83601_MOESM1_ESM.docx]

# Supplementary material

This supplementary material provides the hydrochemical parameters data of shallow and deep groundwater used in the study. Table 1 presents the hydrochemical parameters data for shallow groundwater, while Table 2 presents the hydrochemical parameters data for deep groundwater.

According to the analysis of ion balance errors as assessed by Equation (1), the ion balance error percentages in the analyzed groundwater samples are all found to be less than 7%，that the data are within an acceptable range for ion balance, ensuring the accuracy of the groundwater analysis.

$E=\frac{\sum\text{zm}_{\text{c}}-\sum\text{zm}_{\text{a}}}{\sum\text{zm}_{\text{c}}+\sum\text{zm}_{\text{a}}}\times100$ （1）

Where z is the absolute value of ion valence, m_c_ is the molar concentration of cationic species, m_a_ is the molar concentration of anionic species, and E represents the percentage of ion balance error.

Table 1. Data on shallow groundwater hydrochemical parameters

| Years | Field numbering | Latitude | Longitude | Aquifer group | E | K⁺  (mg/L) | Na⁺  (mg/L) | Ca²⁺  (mg/L) | Mg²⁺  (mg/L) | HCO₃⁻  (mg/L) | CO₃²⁻  (mg/L) | Cl⁻  (mg/L) | SO₄²⁻  (mg/L) | NO₃⁻  (mg/L) | I⁻  (mg/L) | F⁻  (mg/L) | pH | TDS  (mg/L) |
| --- | --- | --- | --- | --- | --- | --- | --- | --- | --- | --- | --- | --- | --- | --- | --- | --- | --- | --- |
| 2014 | G125 | 37.3164 | 115.8958 | Ⅰ | -0.77% | 1.0 | 347.5 | 77.8 | 135.1 | 805.4 | 0 | 314.8 | 403.2 | 0 | 0.152 | 1.61 | 7.6 | 1698.6 |
| 2014 | GT37 | 37.2833 | 115.7922 | Ⅰ | 1.66% | 0.9 | 288.2 | 108.2 | 126.4 | 768.8 | 0 | 290.7 | 320.7 | 0 | 0.203 | 0.65 | 7.23 | 1538.4 |
| 2014 | GT36 | 37.2552 | 115.8624 | Ⅰ | -2.31% | 2.8 | 146.4 | 206 | 115.7 | 466.2 | 0 | 546 | 207.5 | 0 | 0.043 | 0.51 | 7.17 | 1458.5 |
| 2014 | Z110 | 37.4414 | 115.8692 | Ⅰ | 0.43% | 1.9 | 152.5 | 153.1 | 96.7 | 622.4 | 0 | 249.6 | 232.6 | 0 | 0.201 | 0.37 | 7.33 | 1219.5 |
| 2014 | ZT24 | 37.4111 | 115.7122 | Ⅰ | -2.06% | 2.2 | 162.8 | 90.6 | 62.2 | 339.3 | 0 | 207 | 286.8 | 0 | 0.006 | 0.88 | 7.74 | 982.7 |
| 2014 | J133 | 37.6073 | 116.0364 | Ⅰ | 3.83% | 0.9 | 989.1 | 117.8 | 234.8 | 915.3 | 0 | 836.7 | 1204.3 | 0 | 0.279 | 1.84 | 7.05 | 3859.3 |
| 2014 | KK1-2 | 37.5708 | 116.175 | Ⅰ | -0.30% | 1.1 | 625.2 | 220.4 | 264.9 | 544.3 | 0 | 950.1 | 1178.6 | 0 | 0.332 | 1.18 | 7.24 | 3514.6 |
| 2018 | GT37 | 37.2833 | 115.7922 | Ⅰ | -1.00% | 0.29 | 203.16 | 62.19 | 84.24 | 725.61 | 2.97 | 122.14 | 181.1 | 0 | 0.064 | 1.76 | 8.2 | 1034.88 |
| 2018 | G123 | 37.2122 | 115.8078 | Ⅰ | -0.63% | 1.1 | 869.69 | 60.57 | 166.51 | 1055.16 | 2.97 | 491.38 | 1144 | 0.91 | 0.101 | 2.67 | 8.2 | 3280.54 |
| 2018 | G119 | 37.1694 | 115.8536 | Ⅰ | -1.48% | 1.48 | 151.26 | 99.34 | 83.75 | 767.94 | 0.00 | 157.24 | 92.6 | 0 | 0.072 | 0.64 | 7.83 | 989.58 |
| 2018 | G101 | 37.3949 | 116.0528 | Ⅰ | -0.06% | 1.94 | 581.94 | 79.15 | 133.21 | 1133.77 | 0.00 | 315.89 | 613.5 | 0 | 0.163 | 0.94 | 7.64 | 2311.15 |
| 2018 | GT5 | 37.4942 | 116.1275 | Ⅰ | 0.26% | 1.32 | 434.94 | 80.76 | 137.13 | 780.04 | 2.97 | 362.22 | 529.5 | 0 | 0.135 | 1.65 | 8.22 | 1957.15 |
| 2018 | GT15 | 37.4033 | 116.1503 | Ⅰ | -0.18% | 0.55 | 553.46 | 96.91 | 146.92 | 879.81 | 0.00 | 372.04 | 777 | 0 | 0.11 | 0.97 | 7.78 | 2401.14 |
| 2018 | G125 | 37.3164 | 115.8958 | Ⅰ | 2.25% | 1.49 | 277.3 | 83.75 | 140.26 | 651.66 | 0.00 | 292.76 | 366.5 | 1.02 | 0.162 | 1.39 | 7.47 | 1505.71 |
| 2018 | Z124 | 37.2281 | 115.7528 | Ⅰ | -1.94% | 2.69 | 358.34 | 153.45 | 115.09 | 538.16 | 0.00 | 463.30 | 568.8 | 2.53 | 0.049 | 0.54 | 7.25 | 1949.71 |
| 2018 | Z102 | 37.4483 | 115.8053 | Ⅰ | -1.28% | 1.46 | 279.46 | 118.72 | 143.50 | 789.11 | 0.00 | 259.73 | 495 | 0 | 0.067 | 0.76 | 7.27 | 1713.16 |
| 2018 | Z125 | 37.405 | 115.6631 | Ⅰ | -0.97% | 0.87 | 236.34 | 70.26 | 104.81 | 840.50 | 0.00 | 169.88 | 199.1 | 1.12 | 0.076 | 0.91 | 7.43 | 1218.95 |
| 2018 | ZT30 | 37.3061 | 115.7331 | Ⅰ | 0.19% | 1.88 | 388.31 | 177.67 | 122.44 | 780.04 | 2.97 | 491.38 | 428.3 | 2.53 | 0.034 | 0.33 | 8.16 | 2021.47 |
| 2018 | ZT18 | 37.3958 | 115.7275 | Ⅰ | -2.30% | 2.54 | 1380.02 | 222.90 | 369.27 | 1012.84 | 0.00 | 1031.90 | 2872 | 0 | 0.255 | 0.52 | 7.14 | 6402.60 |
| 2018 | GA13 | 37.584 | 115.8659 | Ⅰ | -1.57% | 1.03 | 217.72 | 101.76 | 97.46 | 659.10 | 0.00 | 262.54 | 227.5 | 3.58 | 0.068 | 1.03 | 7.88 | 1261.33 |
| 2018 | Z105 | 37.5053 | 115.8356 | Ⅰ | -0.85% | 1.37 | 376.92 | 112.26 | 154.76 | 846.55 | 0.00 | 379.06 | 509.3 | 1.52 | 0.062 | 0.4 | 7.48 | 1975.47 |
| 2018 | Z103 | 37.3954 | 115.601 | Ⅰ | -3.95% | 0.44 | 333.08 | 87.22 | 84.24 | 822.36 | 0.00 | 181.11 | 433 | 0.94 | 0.328 | 0.64 | 7.43 | 1547.56 |
| 2018 | Z135 | 37.4681 | 115.6942 | Ⅰ | -0.95% | 3.63 | 1260.02 | 722.81 | 673.40 | 837.48 | 0.00 | 2892.11 | 2554.75 | 2.72 | 0.008 | 0.56 | 6.73 | 8541.30 |
| 2018 | ZT32 | 37.5325 | 115.6754 | Ⅰ | 1.15% | 2.26 | 1586.39 | 302.85 | 514.23 | 517.24 | 0.00 | 2101.12 | 2689 | 3.74 | 0.213 | 1.31 | 7.53 | 7475.32 |
| 2018 | Z110 | 37.4414 | 115.8692 | Ⅰ | 1.21% | 8.56 | 260 | 180.91 | 113.13 | 597.04 | 0.00 | 355.79 | 445.6 | 1.53 | 0.176 | 0.34 | 7.6 | 1683.55 |
| 2018 | JT17 | 37.6982 | 116.3837 | Ⅰ | -6.14% | 13.39 | 396.17 | 116.30 | 75.91 | 852.60 | 0.00 | 261.13 | 383.3 | 54.11 | 0.011 | 0.32 | 7.45 | 1740.53 |
| 2018 | J47 | 37.8114 | 116.2794 | Ⅰ | -0.04% | 1.02 | 504.21 | 74.30 | 75.91 | 269.08 | 5.95 | 624.75 | 470.3 | 0 | 0.068 | 2.15 | 8.3 | 1905.36 |
| 2018 | KK1-2 | 37.5708 | 116.175 | Ⅰ | -2.54% | 0.91 | 609.8 | 207.56 | 256.14 | 574.44 | 0.00 | 898.52 | 1232.3 | 1.15 | 0.133 | 0.9 | 7.23 | 3506.36 |
| 2018 | JT21-1 | 37.656 | 116.0348 | Ⅰ | -3.36% | 1.13 | 664.97 | 84.80 | 161.62 | 952.37 | 0.00 | 498.40 | 937.5 | 0 | 0.109 | 2.95 | 7.46 | 2840.09 |
| 2018 | J118 | 37.5386 | 116.045 | Ⅰ | -4.59% | 6.13 | 426.71 | 109.84 | 112.15 | 855.62 | 0.00 | 262.54 | 644.8 | 19.73 | 0.047 | 0.45 | 7.34 | 2024.02 |
| 2022 | 2022QG1 | 37.491582 | 116.129283 | Ⅰ | -1.92% | 1.01 | 449 | 162 | 248 | 849 | 0 | 790 | 637 | 2.57 | 0.096 | 1.36 | 7 | 2686 |
| 2022 | 2022QG2 | 37.403637 | 116.152453 | Ⅰ | -0.17% | 0.83 | 788 | 100 | 199 | 1124 | 0 | 446 | 1188 | 0.27 | 0.321 | 1.4 | 7.4 | 3130 |
| 2022 | 2022QG3 | 37.399213 | 116.064447 | Ⅰ | 1.63% | 1.05 | 433 | 51.6 | 107 | 1131 | 0 | 187 | 261 | 0 | 0.349 | 1.62 | 7.4 | 1554 |
| 2022 | 2022QG4 | 37.25904099 | 115.8679047 | Ⅰ | 5.13% | 0.94 | 1072 | 86 | 238 | 941 | 0 | 830 | 1221 | 0.222 | 0.361 | 2.14 | 7.4 | 3820 |
| 2022 | 2022QG5 | 37.319375 | 115.906785 | Ⅰ | -1.78% | 1.67 | 898 | 64.4 | 161 | 1064 | 0 | 560 | 1146 | 2.01 | 0.4 | 2.46 | 7.4 | 3280 |
| 2022 | 2022QG6 | 37.283697 | 115.793684 | Ⅰ | 1.86% | 0.6 | 226 | 72 | 77.6 | 664 | 0 | 195 | 129 | 0.154 | 0.097 | 1.07 | 7.4 | 1011 |
| 2022 | 2022QG7 | 37.21246 | 115.809194 | Ⅰ | 3.48% | 6.75 | 383 | 93.7 | 140 | 731 | 0 | 317 | 483 | 0.172 | 0.048 | 0.616 | 7.4 | 1776 |
| 2022 | 2022QG8 | 37.169493 | 115.854903 | Ⅰ | 3.17% | 1.42 | 175 | 97.9 | 95.6 | 677 | 0 | 177 | 149 | 0 | 0.101 | 0.707 | 7.3 | 1018 |
| 2022 | 2022QG9 | 37.129612 | 115.8002026 | Ⅰ | 2.42% | 1.54 | 468 | 69.4 | 120 | 886 | 0 | 200 | 579 | 0 | 0.151 | 1.44 | 7.4 | 1855 |
| 2022 | 2022QZ1 | 37.22805556 | 115.7527778 | Ⅰ | -0.86% | 4.4 | 109 | 82.7 | 28.2 | 273 | 0 | 114 | 179 | 0.409 | 0.036 | 0.701 | 7.5 | 642 |
| 2022 | 2022QZ2 | 37.50527778 | 115.8355556 | Ⅰ | -0.05% | 1.03 | 379 | 144 | 159 | 855 | 0 | 446 | 453 | 10.5 | 0.113 | 0.681 | 7.1 | 2001 |
| 2022 | 2022QZ3 | 37.44138889 | 115.8691667 | Ⅰ | -2.95% | 8.55 | 251 | 176 | 99.2 | 545 | 0 | 353 | 504 | 2.51 | 0.107 | 0.486 | 7.1 | 1602 |
| 2022 | 2022QZ4 | 37.55017222 | 115.8817944 | Ⅰ | 0.45% | 2.78 | 495 | 155 | 122 | 966 | 0 | 514 | 400 | 4.79 | 0.277 | 0.969 | 7.2 | 2102 |
| 2022 | 2022QZ5 | 37.30611111 | 115.7330556 | Ⅰ | 0.38% | 2.34 | 428 | 162 | 129 | 653 | 0 | 594 | 460 | 0.707 | 0.14 | 0.543 | 7.2 | 2079 |
| 2022 | 2022QZ6 | 37.58397222 | 115.8659167 | Ⅰ | 0.57% | 1.65 | 268 | 138 | 159 | 664 | 0 | 488 | 318 | 0.02 | 0.03 | 1.31 | 7.3 | 1684 |
| 2022 | 2022QZ7 | 37.39583333 | 115.7275 | Ⅰ | 0.02% | 2.1 | 942 | 147 | 275 | 971 | 0 | 768 | 1601 | 0.105 | 0.4 | 0.995 | 7.4 | 4130 |
| 2022 | 2022QZ8 | 37.44602222 | 115.8614583 | Ⅰ | 4.86% | 1.1 | 379 | 98.5 | 136 | 778 | 0 | 253 | 476 | 0.11 | 0.241 | 1.79 | 7.3 | 1704 |
| 2022 | 2022QZ9 | 37.44833333 | 115.8052778 | Ⅰ | -1.51% | 1.21 | 337 | 134 | 155 | 746 | 0 | 301 | 685 | 0.129 | 0.105 | 0.872 | 7.1 | 1958 |
| 2022 | 2022QZ10 | 37.46805556 | 115.6941667 | Ⅰ | 2.66% | 2.78 | 394 | 125 | 67.3 | 674 | 0 | 284 | 323 | 24.8 | 0.011 | 0.588 | 7.6 | 1605 |
| 2022 | 2022QJ1 | 37.530102 | 116.048463 | Ⅰ | 2.45% | 6.08 | 536 | 162 | 168 | 741 | 27 | 481 | 737 | 34.8 | 0.003 | 0.445 | 8.2 | 2582 |
| 2022 | 2022QJ2 | 37.570536 | 116.178473 | Ⅰ | 5.69% | 0.94 | 628 | 217 | 272 | 561 | 0 | 861 | 1023 | 0.617 | 0.314 | 1.09 | 7.2 | 3260 |
| 2022 | 2022QJ3 | 37.661822 | 116.032903 | Ⅰ | 0.16% | 0.97 | 536 | 69 | 117 | 868 | 0 | 351 | 578 | 0 | 0.109 | 2.72 | 7.4 | 2047 |
| 2022 | 2022QJ4 | 37.533611 | 116.264969 | Ⅰ | 1.70% | 0.7 | 477 | 219 | 146 | 647 | 0 | 512 | 806 | 8.1 | 0 | 0.458 | 7.3 | 2497 |
| 2022 | 2022QJ5 | 37.698804 | 116.37469 | Ⅰ | 1.62% | 12 | 407 | 170 | 126 | 810 | 0 | 378 | 512 | 16.5 | 0 | 0.454 | 7.2 | 2058 |

Table 2. Data on deep groundwater hydrochemical parameters

| Years | Field numbering | Latitude | Longitude | Aquifer group | E | K⁺  (mg/L) | Na⁺  (mg/L) | Ca²⁺  (mg/L) | Mg²⁺  (mg/L) | HCO₃⁻  (mg/L) | CO₃²⁻  (mg/L) | Cl⁻  (mg/L) | SO₄²⁻  (mg/L) | NO₃⁻  (mg/L) | I⁻  (mg/L) | F⁻  (mg/L) | pH | TDS  (mg/L) |
| --- | --- | --- | --- | --- | --- | --- | --- | --- | --- | --- | --- | --- | --- | --- | --- | --- | --- | --- |
| 2014 | G306 | 37.3203 | 115.9011 | Ⅲ | 5.54% | 0.8 | 227.8 | 12.8 | 5.3 | 371 | 21.6 | 62.4 | 91.5 | 0 | 0.295 | 2.65 | 8.99 | 625 |
| 2014 | G301 | 37.4036 | 116.1508 | Ⅲ | 5.07% | 0.5 | 260.5 | 7.2 | 7.8 | 436.2 | 11.7 | 82.3 | 77 | 0 | 0.105 | 2.1 | 8.42 | 668.7 |
| 2014 | Z43 | 37.2826 | 115.7136 | Ⅲ | 0.08% | 0.4 | 192.9 | 8 | 6.3 | 331.9 | 7.2 | 68.1 | 88.7 | 0 | 0.201 | 1.83 | 8.36 | 553.3 |
| 2014 | Z30-1 | 37.3186 | 115.6375 | Ⅲ | -0.70% | 0.4 | 194.7 | 19.2 | 12.6 | 168.4 | 4.8 | 143.2 | 179.4 | 0 | 0.101 | 1.55 | 8.29 | 640.8 |
| 2014 | KS1 | 37.57 | 116.1772 | Ⅲ | 2.47% | 0.4 | 242.8 | 15.2 | 16.5 | 324.6 | 7.2 | 131.9 | 142.4 | 0 | 0.214 | 1.9 | 8.34 | 734.6 |
| 2014 | J30 | 37.5658 | 116.1133 | Ⅲ | 0.70% | 0.5 | 243.3 | 10.4 | 5.3 | 285.6 | 9.6 | 137.6 | 131.2 | 0 | 0.101 | 1.9 | 8.42 | 697.3 |
| 2018 | G303-1 | 37.2158 | 115.8156 | Ⅲ | 1.50% | 0.56 | 223.94 | 5.65 | 8.82 | 423.27 | 14.87 | 56.16 | 83.80 | 0.00 | 0.12 | 3.58 | 8.44 | 624.98 |
| 2018 | G307 | 37.4753 | 116.0031 | Ⅲ | 1.01% | 0.42 | 193.34 | 4.85 | 5.39 | 308.39 | 11.89 | 64.58 | 93.30 | 0.00 | 0.12 | 2.10 | 8.39 | 544.20 |
| 2018 | G306 | 37.3203 | 115.9011 | Ⅲ | 5.74% | 0.52 | 235.95 | 5.65 | 5.88 | 387.19 | 8.72 | 61.63 | 78.70 | 0.00 | 0.25 | 2.81 | 8.20 | 609.01 |
| 2018 | GX1 | 37.3974 | 116.0449 | Ⅲ | 0.07% | 0.51 | 227.25 | 6.46 | 8.82 | 411.18 | 8.92 | 80.02 | 83.70 | 0.00 | 0.14 | 3.66 | 8.34 | 639.15 |
| 2018 | Z31 | 37.5018 | 115.8378 | Ⅲ | -2.39% | 0.50 | 210.36 | 16.96 | 11.26 | 238.85 | 8.92 | 155.84 | 144.00 | 0.00 | 0.11 | 2.69 | 8.34 | 684.66 |
| 2018 | Z312-1 | 37.5889 | 115.8447 | Ⅲ | -0.20% | 0.57 | 218.15 | 8.88 | 5.39 | 211.64 | 8.92 | 123.55 | 163.70 | 0.00 | 0.04 | 1.31 | 8.33 | 650.21 |
| 2018 | Z30 | 37.3186 | 115.6375 | Ⅲ | 1.78% | 0.43 | 193.83 | 12.11 | 7.35 | 196.52 | 5.95 | 119.33 | 128.50 | 0.00 | 0.09 | 1.19 | 8.29 | 581.60 |
| 2018 | Z313 | 37.3986 | 115.6956 | Ⅲ | 3.84% | 0.55 | 206.30 | 11.31 | 8.82 | 316.26 | 8.72 | 72.84 | 104.20 | 0.00 | 0.16 | 2.59 | 8.34 | 589.16 |
| 2018 | Z314 | 37.2839 | 115.7094 | Ⅲ | 6.09% | 0.64 | 220.30 | 8.08 | 6.86 | 319.21 | 11.63 | 72.84 | 95.10 | 0.00 | 0.21 | 2.30 | 8.36 | 592.19 |
| 2018 | J57 | 37.8436 | 116.3356 | Ⅲ | 2.43% | 0.44 | 279.48 | 16.15 | 17.63 | 293.27 | 2.97 | 185.32 | 170.50 | 0.00 | 0.06 | 3.50 | 8.20 | 836.91 |
| 2018 | J51 | 37.5331 | 116.045 | Ⅲ | -1.65% | 0.35 | 259.13 | 10.50 | 7.35 | 335.60 | 8.92 | 141.80 | 150.10 | 0.00 | 0.07 | 3.60 | 8.33 | 763.48 |
| 2018 | J301 | 37.8039 | 116.425 | Ⅲ | -1.52% | 0.51 | 247.82 | 7.27 | 4.90 | 362.81 | 8.92 | 91.26 | 124.00 | 0.00 | 0.11 | 2.24 | 8.34 | 681.66 |
| 2018 | KS1 | 37.57 | 116.1772 | Ⅲ | -0.13% | 0.38 | 223.57 | 10.50 | 10.77 | 350.71 | 11.89 | 94.06 | 125.20 | 0.00 | 0.12 | 3.14 | 8.37 | 667.28 |
| 2022 | 2022SG1 | 37.480055 | 116.000955 | Ⅲ | 3.11% | 0.44 | 205 | 6.43 | 4.6 | 329 | 0 | 61.6 | 86.8 | 0.031 | 0.198 | 2.22 | 8.3 | 504 |
| 2022 | 2022SG2 | 37.400837 | 116.064897 | Ⅲ | 3.73% | 0.46 | 258 | 9.13 | 11.5 | 379 | 17 | 114 | 103 | 0 | 0.315 | 3.58 | 8.2 | 661 |
| 2022 | 2022SG3 | 37.31928 | 115.906793 | Ⅲ | -0.01% | 0.63 | 213 | 7.09 | 8.25 | 418 | 16 | 58.1 | 77 | 0.156 | 0.247 | 4.05 | 8.3 | 558 |
| 2022 | 2022SG4 | 37.215155 | 115.809726 | Ⅲ | 2.51% | 0.68 | 263 | 9.78 | 11.8 | 408 | 7 | 105 | 119 | 0 | 0.261 | 3.41 | 8.1 | 701 |
| 2022 | 2022SZ1 | 37.31861111 | 115.6375 | Ⅲ | -1.52% | 0.57 | 190 | 14.8 | 8.77 | 191 | 10 | 127 | 156 | 0.033 | 0.08 | 1.23 | 8.2 | 578 |
| 2022 | 2022SZ2 | 37.39861111 | 115.6955556 | Ⅲ | 2.78% | 0.38 | 176 | 7.59 | 5.43 | 266 | 36 | 55.9 | 95.4 | 0 | 0.226 | 2.4 | 8.3 | 462 |
| 2022 | 2022SZ3 | 37.58888889 | 115.8447222 | Ⅲ | -1.34% | 4.98 | 80.3 | 53.3 | 31.1 | 152 | 8 | 86.2 | 187 | 2.89 | 0.009 | 0.539 | 8.1 | 521 |
| 2022 | 2022SZ4 | 37.28394444 | 115.7094444 | Ⅲ | 3.64% | 0.66 | 317 | 11.6 | 20.2 | 472 | 13 | 144 | 143 | 0 | 0.32 | 3.76 | 8 | 848 |
| 2022 | 2022SZ5 | 37.50177778 | 115.8377778 | Ⅲ | 0.45% | 0.41 | 189 | 11.7 | 8.27 | 216 | 11 | 125 | 107 | 0 | 0.102 | 2.27 | 8.2 | 531 |
| 2022 | 2022SJ1 | 37.530441 | 116.046103 | Ⅲ | 2.08% | 0.34 | 195 | 8.03 | 6.93 | 301 | 20 | 69.1 | 98.8 | 0 | 0.215 | 2.86 | 8.2 | 501 |
| 2022 | 2022SJ2 | 37.570167 | 116.178727 | Ⅲ | 3.81% | 0.67 | 320 | 25.5 | 36.7 | 329 | 9 | 265 | 190 | 0 | 0.24 | 2.96 | 8 | 982 |
| 2022 | 2022SJ3 | 37.60984 | 116.302862 | Ⅲ | 1.61% | 0.27 | 213 | 7.33 | 7.23 | 331 | 15 | 82.5 | 95.5 | 0 | 0.244 | 3.38 | 8.3 | 548 |
| 2022 | 2022SJ4 | 37.69899249 | 116.3716888 | Ⅲ | -2.11% | 0.35 | 252 | 10.9 | 13.2 | 346 | 13 | 150 | 142 | 0.683 | 0.26 | 4.08 | 8.1 | 732 |
| 2022 | 2022SJ5 | 37.811559 | 116.280961 | Ⅲ | -0.18% | 0.49 | 275 | 16.4 | 17.4 | 280 | 14 | 198 | 188 | 0 | 0.058 | 3.53 | 8 | 821 |
| 2022 | 2022SJ6 | 37.803084 | 116.412428 | Ⅲ | 3.39% | 0.52 | 279 | 8.63 | 7.61 | 330 | 12 | 135 | 143 | 0 | 0.079 | 3.24 | 8.2 | 724 |
